# Dectin-3 Deficiency Promotes Colitis Development due to Impaired Antifungal Innate Immune Responses in the Gut

**DOI:** 10.1371/journal.ppat.1005662

**Published:** 2016-06-09

**Authors:** Tingting Wang, Deng Pan, Zhicheng Zhou, Yun You, Changying Jiang, Xueqiang Zhao, Xin Lin

**Affiliations:** 1 Department of Molecular and Cellular Oncology, The University of Texas, MD Anderson Cancer Center, Houston, Texas, United States of America; 2 The State Key Laboratory of Pharmaceutical Biotechnology, Division of Immunology, Medical School, Nanjing University, Nanjing, China; 3 Cancer Biology Program, The University of Texas, Graduate School of Biomedical Sciences, Houston, Texas, United States of America; 4 Institute for Immunology, Tsinghua University School of Medicine, Beijing, China; University of Pittsburgh, UNITED STATES

## Abstract

Interactions between commensal fungi and gut immune system are critical for establishing colonic homeostasis. Here we found that mice deficient in Dectin-3 (*Clec4d* ^*-/-*^), a C-type lectin receptor that senses fungal infection, were more susceptible to dextran sodium sulfate (DSS)-induced colitis compared with wild-type mice. The specific fungal burden of *Candida* (*C*.) *tropicalis* was markedly increased in the gut after DSS treatment in *Clec4d* ^*-/-*^ mice, and supplementation with *C*. *tropicalis* aggravated colitis only in *Clec4d* ^*-/-*^ mice, but not in wild-type controls. Mechanistically, Dectin-3 deficiency impairs phagocytic and fungicidal abilities of macrophages, and *C*. *tropicalis*-induced NF-κB activation and cytokine production. The conditioned media derived from Dectin-3-deficient macrophages were defective in promoting tissue repairing in colonic epithelial cells. Finally, anti-fungal therapy was effective in treating colitis in *Clec4d* ^*-/-*^ mice. These studies identified the role of Dectin-3 and its functional interaction with commensal fungi in intestinal immune system and regulation of colonic homeostasis.

## Author Summary

C-type lectin receptors (CLRs) comprise a diverse family of soluble and trans-membrane proteins that function as pattern recognition receptors (PRRs). Dectin-3 (also known as MCL/CLECSF8/Clec4d), a myeloid cell-specific CLR family member, could recognize bacterial and fungal components and induce intracellular signaling pathways that regulate the immune response. Although investigators have explored the role of Dectin-3 in systemic immunity, its function in the gastrointestinal immune system is not clear. Using a dextran sodium sulfate (DSS)-induced colitis mice model, we show here Dectin-3-deficient mice were more susceptible to DSS-induced colitis compared with wild-type mice. The specific fungal burden of a commensal fungi *C*. *tropicalis* was markedly increased in the gut after DSS treatment in Dectin-3-deficient mice, and antifungal therapy could effectively protect these mice from colitis. Taken together, we demonstrate the important function of Dectin-3 and its functional interaction with commensal fungi in intestinal immune responses and regulation of colonic homeostasis.

## Introduction

Inflammatory bowel disease (IBD), mainly Crohn disease and ulcerative colitis, is a chronic inflammatory disorder of the gut. Extensive studies have suggested that the etiology of IBD involves environmental and genetic factors that lead to dysfunction of the epithelial barrier, with consequent deregulation of the mucosal immune system and responses to microbiota [1,2]. Therefore, interactions between the commensal microbiota and gut immune system are critical for establishing colonic epithelial homeostasis.

Mammalian gastrointestinal tract is colonized with multiple microbial communities, including bacteria, fungi and viruses. Although the vast majority of studies on commensal microbiota have focused on bacteria, commensal fungi were reported [3] and were linked with a number of gastrointestinal disease including IBD [4], irritable bowel syndrome [5], gastric ulcers[6] and chemotherapy-induced enteric disorders [7]. Gastrointestinal tract of healthy individuals contains 66 fungal genera and 184 fungal species, with *Candida* as the dominant fungal genera [8]. These fungi could become pathogenic as the result of a change in the environment, for example the loss or reduction of bacterial or suppression of immune system. Recently, Iliev et al. showed that dextran sodium sulfate (DSS) treatment allows pathogenic fungi to invade the intestinal wall and that Dectin-1 plays an important role in protecting the host from colitis [9]. Therefore, deficiencies in genes involved in innate and adaptive immune pathways may lead to disorders characterized by intestinal manifestations and loss of microbial diversity [10]. However, a recent study by Tang et al. showed that suppression of Dectin-1 signaling protects mice from experimental colitis by decreasing S100A8 and S100A9 antimicrobial peptide production [11], which allows the overgrowth of *L*. *murinus* that trigger T regulatory cell expansion in the gut. These findings suggest that Dectin-1 can play opposite roles in DSS-induced colitis dependent on the microbial community in the gut.

Several mammalian C-type lectin receptors (CLRs), including Dectin-1, Dectin-2, Dectin-3, and Mincle, function as pattern recognition receptors sensing fungal infections and inducing multiple signaling cascades, which lead to expression of various pro-inflammatory cytokines and antimicrobial proteins [12–18]. Specifically, Dectin-1 recognizes β-glucans on the surface of fungal yeast cells, whereas Dectin-2 recognizes α-mannan on the surface of fungal hyphae. Dectin-3, a CLR also known as MCL/CLECSF8/Clec4d, functions as a pattern recognition receptor for sensing fungal infections by recognizing α-mannans [19,20]. Our previous data indicated that Dectin-3 forms a heterodimeric complex with Dectin-2, which recognizes α-mannans and has greater sensitivity in sensing *Candida albicans* infections than either the Dectin-2 or Dectin-3 homodimer, leading to potent activation of NF-κB–dependent antifungal immune responses [12]. Dectin-3 is expressed by peripheral blood neutrophils, monocytes, and various subsets of dendritic cells [20]. Recognition of these cell wall components by CLRs induces the Syk/Caspase recruitment domain 9 (CARD9)/NF-κB–dependent signaling pathway [21], leading to production of inflammatory cytokines in innate immune cells and participating in antifungal responses. Furthermore, the induced pro-inflammatory cytokines regulate Th17 and Th1 cell differentiation. Subsequently, cytokines produced by Th17 and Th1 cells activate neutrophils and macrophages that mediate the clearance of infected fungi in vivo. Although investigators have explored the role of Dectin-3 in systemic immunity, its function in the gastrointestinal immune system has yet to be investigated.

In this study, we have identified the role of Dectin-3 and its functional interaction with commensal fungi in intestinal immune responses and regulation of colonic homeostasis. We found that Dectin-3-deficient (*Clec4d* ^*-/-*^) mice were more susceptible to DSS-induced colitis compared with wild-type mice. The specific fungal burden of *C*. *tropicalis* was markedly increased in the gut after DSS treatment in *Clec4d* ^*-/-*^ mice. Mechanistically, absence of Dectin-3 impairs the phagocytic and fungicidal abilities of macrophages. Dectin-3 is also required for *C*. *tropicalis*-induced CARD9/Bcl10 complex formation and NF-κB activation, which in turn induce tissue-repairing program in colonic epithelial tissues.

## Results

### Dectin-3-deficient mice are susceptible to DSS-induced colitis

**Fig 1:**
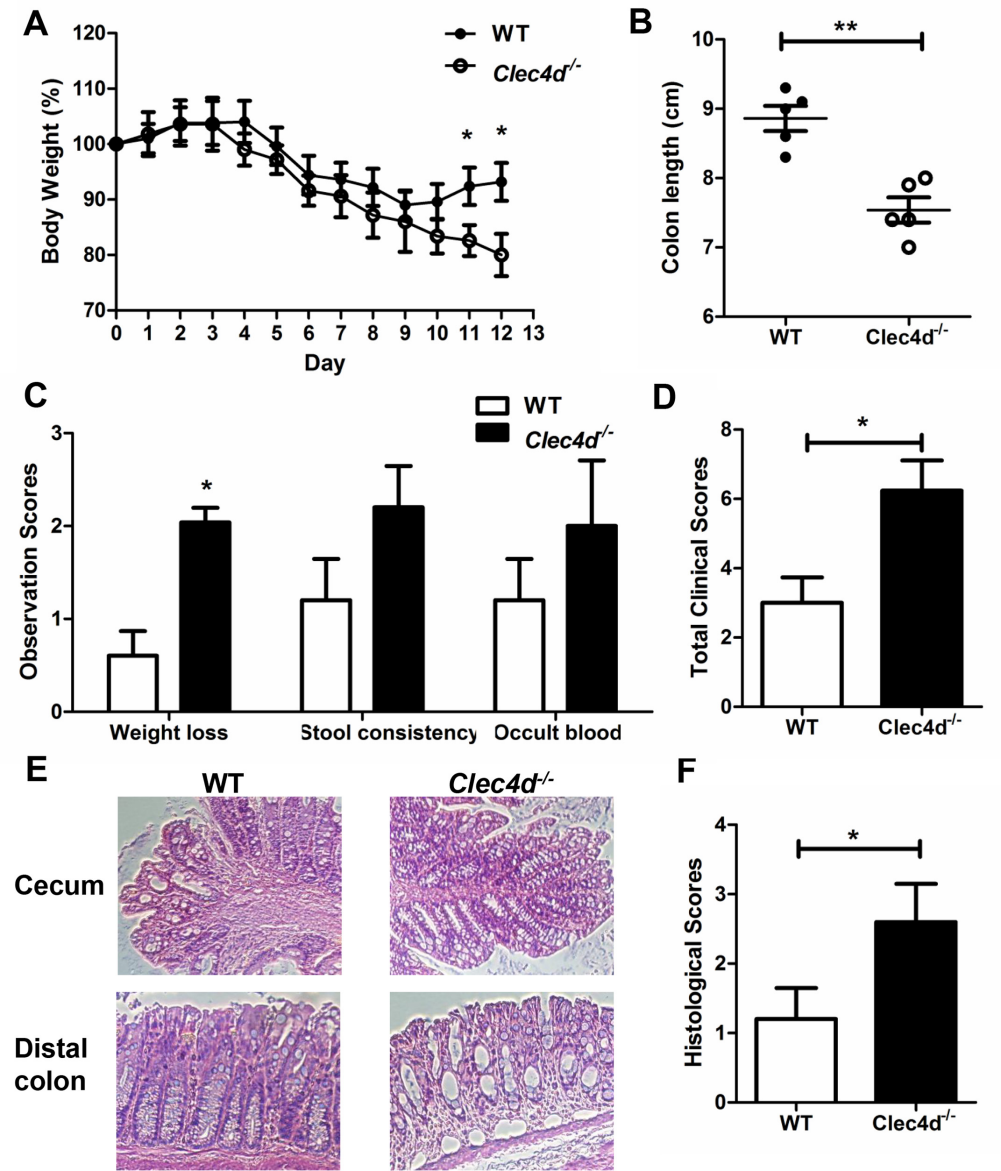
Mice lacking Dectin-3 expression exhibited severe colitis. Wild-type (WT) and *Clec4d* ^*-/-*^ single housed mice (*n* = 5 per group) were given 2.5% DSS for 7 days and then water for an additional 4 days. Mice were sacrificed on day 12. The progress and severity of colitis in the mice were assessed by measuring body weight during treatment (A) and colon length (B). (C and D) Clinical colitis scores and were calculated based on weight loss, stool consistency, and colon bleeding. (E) Colon sections obtained from the mice were stained with H&E. (F) Colitis severity was accessed according to histology score. Data represent one of two independent experiments. Error bars, SD. *P < 0.05; **P < 0.01.

Although recent studies indicate that Dectin-3 plays important roles in innate immune responses against fungal and bacterial infections [12,22–24], the role of Dectin-3 in mucosal immunity has not been examined. To determine the role of Dectin-3 in mucosal immune responses, we first check the body weight and colons for spontaneous colitis. Both female and male *Clec4d* ^*-/-*^ mice have normal increase of body weight compared with wild-type mice (S1A and S1B Fig). On histological examination, intestinal epithelial cell appeared normal and no observed spontaneous colitis was found in *Clec4d* ^*-/-*^ mice (S1C Fig). We therefore induce intestinal injury and inflammation using DSS colitis model. After treatment with 2.5% DSS for 7 days and being given water for an additional 4 days, *Clec4d* ^*-/-*^ mice had greater weight loss, shorter colon lengths, and higher clinical scores than wild-type mice (Fig 1A–1D). Similar results were found in another independent experiment (S1D Fig). *Clec4d* ^*-/-*^ mice also exhibited increased mucosal erosion, inflammatory cell infiltration, crypt destruction and loss of goblet cells in the colon, compared with wild-type mice (Fig 1E and 1F). Similar results were obtained comparing co-housed animals (S1E Fig). These data suggest that *Clec4d* ^*-/-*^ mice were more susceptible to DSS-induced colitis.

### Dectin-3-deficient mice showed impaired immune responses after DSS administration

**Fig 2:**
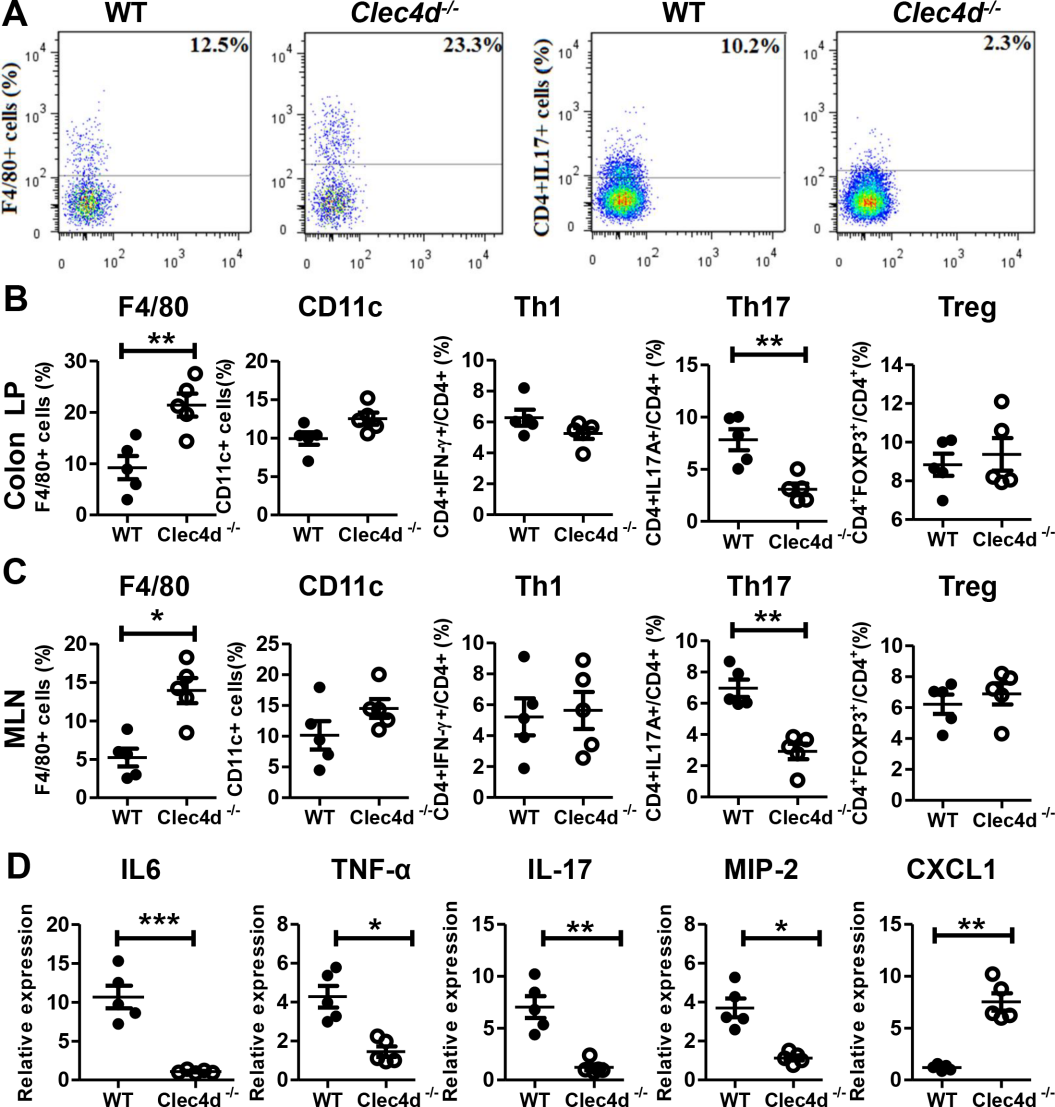
Dectin-3-deficient mice showed impaired immune responses after DSS administration. WT and *Clec4d* ^*−/−*^ mice (*n* = 5 per group) were treated as described in Fig 1. Colonic lamina propria cells and mesenteric lymph nodes cells were separated. (A) Representative image of F4/80^+^ cells and CD4^+^IL17^+^ cells /CD4^+^ cells in the colonic lamina propria (LP) of WT and *Clec4d* ^*−/−*^ mice. (B and C) Frequency of innate and adaptive immune cells including, F4/80^+^ cells, CD11c^+^ cells, CD4^+^IFNγ^+^ cells/CD4^+^ cells, CD4^+^IL17^+^ cells /CD4^+^ cells and CD4^+^CD25^+^Foxp3^+^ cells/CD4^+^ cells were detected in mesenteric lymph nodes (MLN) and colonic LP using flow cytometry. (D) Cytokine expression levels in colonic LP cells were assayed using qPCR. Data represent one of two independent experiments. Error bars, SD. * *P*<0.05, ** *P*<0.01, *** *P*<0.001.

We then detected innate and adaptive immune cells in the mesenteric lymph nodes (MLN) and colonic lamina propria (LP) of mice given DSS. In a comparison with wild-type mice, we found that more macrophages were recruited to MLN and colonic LP in *Clec4d* ^*-/-*^ mice, whereas fewer Th17 cells were located in the MLN and LP of *Clec4d* ^*-/-*^ mice (Fig 2A–2C).

We also measured colonic expression levels of both cytokines and chemokines using qRT-PCR. No significant differences were observed between wild-type and *Clec4d* ^*-/-*^ mice in colitis induction stage (S2A Fig). After water recovery stage, the expression of IL-6, IL-17a, TNF-α, and macrophage inflammatory protein-2 (MIP-2) was lower in *Clec4d* ^*-/-*^ mice than in wild-type mice (Fig 2D). We also found increased expression of chemokine CXCL1 in the colons of *Clec4d* ^*-/-*^ mice than in wild-type. Similar results were found in the protein production levels of IL-6, TNF-α, IL-17a, and MIP-2 in colonic LP cells supernatant (S2B Fig). The colonic expression of IL-10, IL-22, IL-1β and IFN-γ were increased similarly in wild-type and *Clec4d* ^*-/-*^ mice (S2C Fig). Systemic IL-6, TNF-α, and IL-17a levels in serum were similar in wild-type and *Clec4d* ^*-/-*^ mice after DSS treatment (S2D Fig), suggesting that these responses were specific for the intestinal environment. Furthermore, we found decreased expression of IL-6 and IL-17a and increased expression of CXCL1 in MLNs of *Clec4d* ^*-/-*^ compared with wild-type (S2E Fig). IL-6 protects the intestinal epithelium injury by regulating Stat3 signaling [25]. Therefore, the defect in IL-6 expression observed in *Clec4d* ^*-/-*^ mice also may play a role in impaired epithelial restitution.

### Supplementation with *C*. *tropicalis* aggravates colitis in Dectin-3-deficient mice

**Fig 3:**
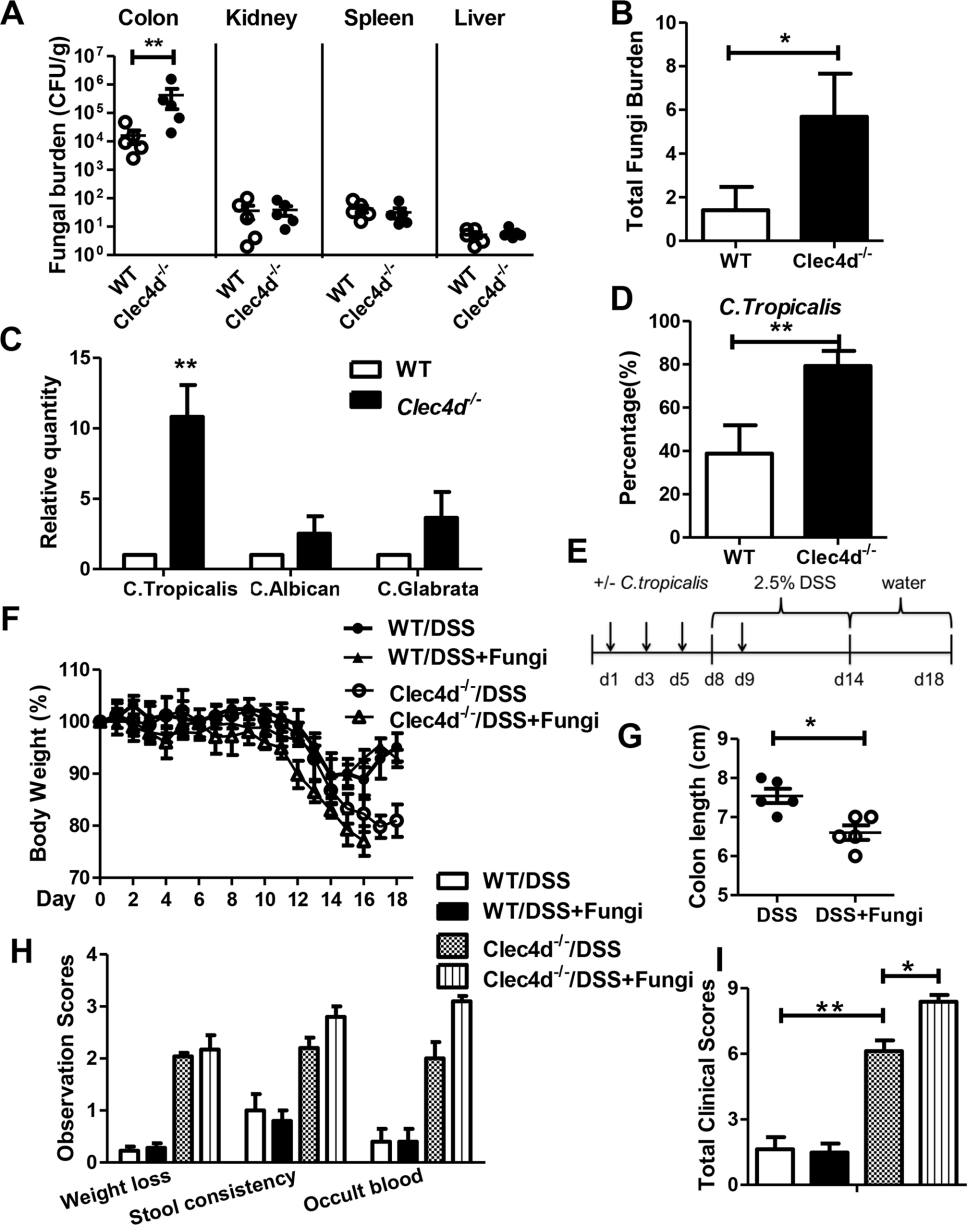
*C*. *tropicalis* supplementation aggravates colitis in Dectin-3-deficient mice. (A)WT and *Clec4d* ^*-/-*^ mice (n = 5 per group) were treated with as described in Fig 1. Total fungal burdens in the colon, kidney, spleen, and liver were detected using a CFU assay. (B) Quantitative analysis of total fungal burden in the feces of WT and *Clec4d* ^*-/-*^ mice using qPCR. (C) Quantitative analysis of *C*. *tropicalis*, *C*.*albicans and C*.*glabrata* in the feces of WT and *Clec4d* ^*-/-*^ mice at the end of mice model using qPCR. (D) The ratio of *C*.*tropicalis* to total fungus was calculated using qPCR. (E) WT and *Clec4d* ^*-/-*^ mice (n = 5 per group) underwent supplementation with or without four doses of *C*. *tropicalis* and then given 2.5% DSS for 7 days and water for an additional 4 d. (F and G) Severity of colitis were assessed by measuring body weights and colon lengths of each mice. (H-I) Disease severity was assessed using clinical colitis scores. Data represent one of two independent experiments. Error bars, SD. *P < 0.05; **P < 0.01.

Since Dectin-3 is involved in anti-fungal immunity [12], we then examined the fungal burden in the colon. The basal level of total fungal burden in colons has no difference between *Clec4d* ^*-/-*^ and wild-type mice (S3A Fig). After DSS treatment, the total fungal burden in the colon was markedly higher in *Clec4d* ^*-/-*^ mice than in wild-type mice (Fig 3A and 3B). A previous study proved that *Candida* is the major intestinal fungal genus in mice [9]. Therefore, we quantified the relative levels of *C*. *tropicalis*, *C*. *albicans*, and *C*. *glabrata* in colon using quantitative PCR. We found that only *C*. *tropicalis* increased in *Clec4d* ^*-/-*^ mice during colitis (Fig 3C). The proportion of *C*.*tropicalis* in total fungi was increased after DSS treatment (Fig 3D and S3B Fig). Since Dectin-3 also involved in innate immune responses against bacterial infections, which may affect colitis progression, we then detected the bacteria burden before and after DSS treatment. As shown in S3C Fig, no difference of total bacteria burden was found between *Clec4d* ^*-/-*^ and wild-type mice.

Given that *C*. *tropicalis* is an opportunistic pathogen, we further analyzed its role in the development of colitis in *Clec4d* ^*-/-*^ mice. We supplemented mice with *C*. *tropicalis* and gave them DSS as outlined in Fig 3E. This supplement could increase the fungal burden in both *Clec4d* ^*-/-*^ and wild-type mice (S3D Fig). As shown in Fig 3F, the body weights of mice given *C*. *tropicalis* and DSS decreased by 20% on day 15. We had to sacrifice the mice on day 16 according to our protocol. We found shorter colon length and more severe clinical scores in *Clec4d* ^*-/-*^ mice supplemented with *C*. *tropicalis* than those in *Clec4d* ^*-/-*^ mice (Fig 3F–3I). However, the pathological changes of distal colon and cecum were similar in *Clec4d* ^*-/-*^ mice supplemented with or without *C*. *tropicalis* (S3E Fig). In contrast, supplementation with *C*. *tropicalis* did not aggravate colitis in WT mice.

To exclude the effect of fungal supplementation alone on colitis, we supplemented mice with *C*. *tropicalis* alone without DSS, and found that *C*. *tropicalis* could not induce colitis in both wild-type and *Clec4d* ^*-/-*^ mice without DSS treatment (S4A–S4C Fig).

### Dectin-3–deficient macrophages had impaired phagocytic and fungicidal abilities

**Fig 4:**
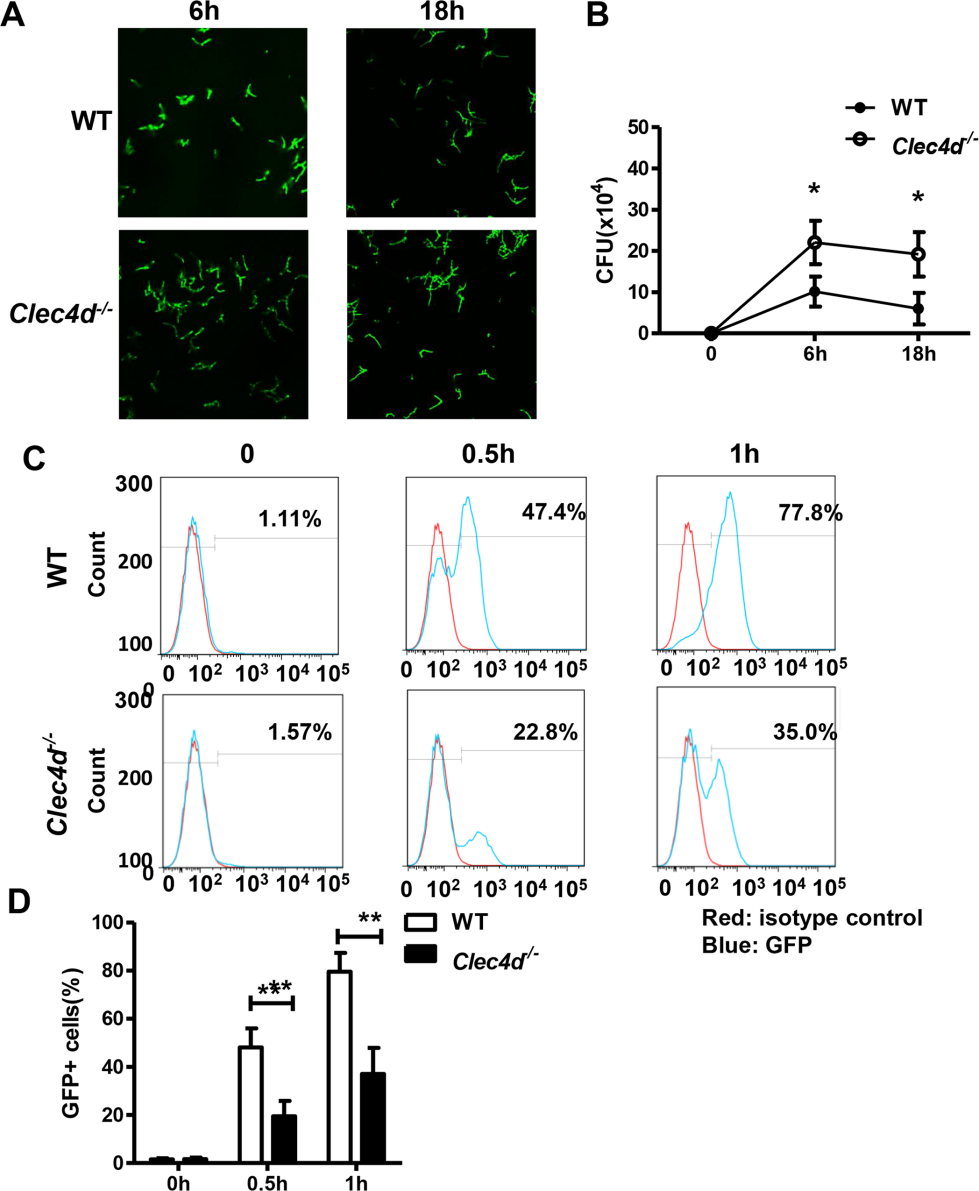
Dectin-3–deficient macrophages had impaired phagocytic and fungicidal abilities. (A and B) GFP-*C*. *tropicalis* (5×10^6^) were added onto 1×10^6^ BMDMs, and incubated at 37°C for 1 hour. Wells were washed and fresh media containing fluconazole (300 μg/ml) was added. At 6 hours and 18 hours, *C*. *tropicalis* CFU inside BMDMs were photographed and calculated by plating on YPD agar. (C and D) GFP-*C*. *tropicalis* (5×10^6^) were added onto 1×10^6^ BMDMs, and incubated at 37°C for indicated times. GFP^+^ BMDMs were calculated by flow cytometry. Data represent one of three independent experiments. Error bars, SD. *P < 0.05; **P < 0.01.

The above *C*.*tropicalis* supplement experiment suggests an impaired fungal killing ability of Dectin-3 deficient mice. To determine the role of Dectin-3 in antifungal immunity, we examined phagocytic and fungicidal abilities in primary macrophages obtained from bone marrow (BMDMs) following challenge with *C*. *tropicalis*. We found that wild-type BMDMs were able to limit the intracellular replication of *C*. *tropicalis*, whereas *Clec4d* ^*-/-*^ BMDMs had a much larger fungal load (Fig 4A). CFU assays demonstrated an increased number of viable yeasts recovered from *Clec4d* ^*-/-*^ macrophages (Fig 4B). The difference between wild-type and *Clec4d* ^*-/-*^ macrophages in killing the phagocytosed *C*. *tropicalis*, was not due to a difference in phagocytosis by these macrophages, as wild-type BMDMs showed a higher phagocytosis ability (Fig 4C and 4D) than *Clec4d* ^*-/-*^ BMDMs. Therefore, Dectin-3 may have an important role in the initial fungal killing process and may prevent the phagocytosed *C*. *tropicalis* from escaping from phagosomes.

### Dectin-3–deficient macrophages are defective in *C*. *tropicalis*-induced NF-κB activation

**Fig 5:**
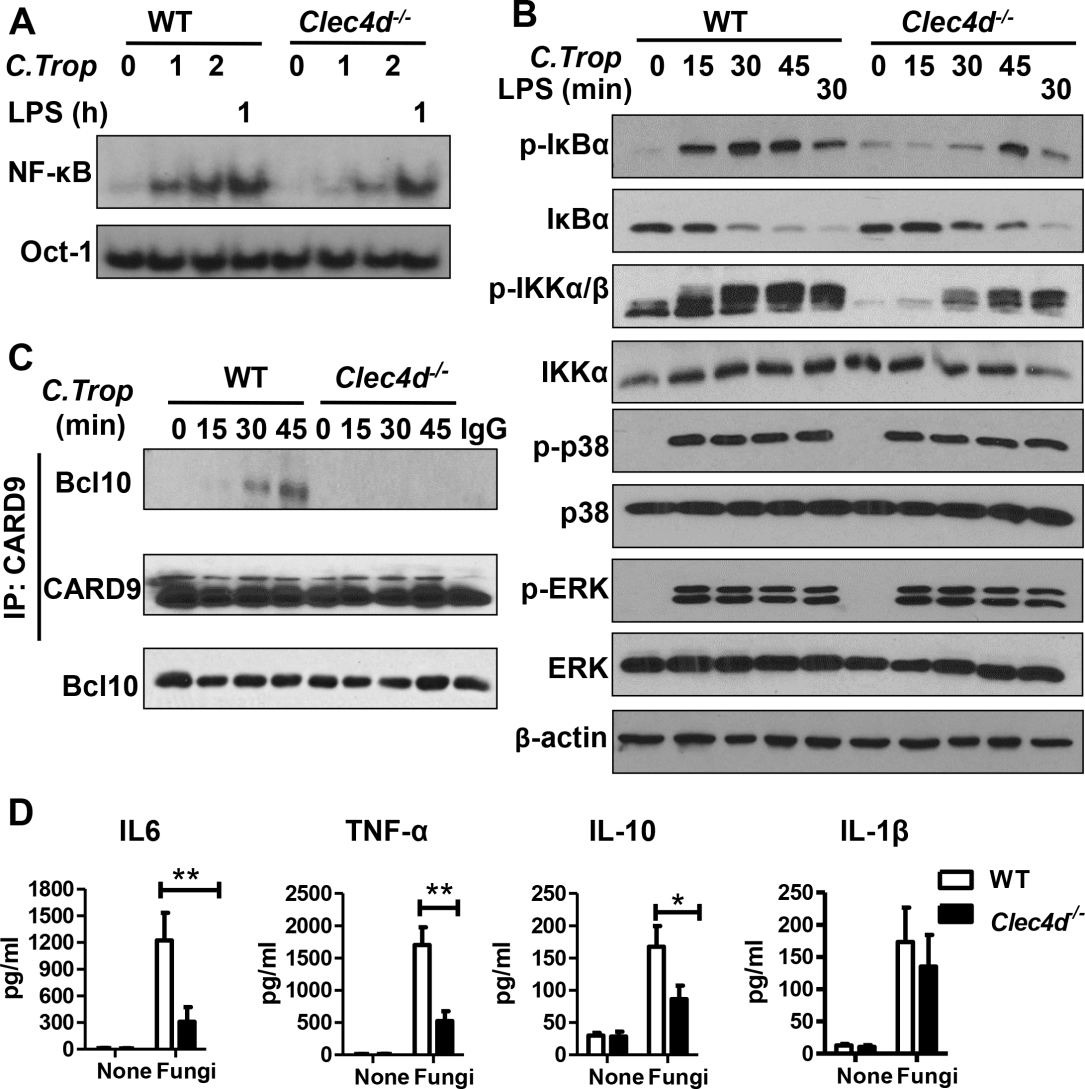
Dectin-3–deficient macrophages were defective in *C*. *tropicalis*-induced NF-κB activation. (A) WT and *Clec4d* ^*-/-*^ BMDMs were stimulated with *C*. *tropicalis* (MOI = 1) or LPS (100 ng/ml) for the indicated times. Nuclear extracts were prepared from these cells and subjected to electrophoretic mobility shift assay using ^32^P-labeled NF-κB and Oct-1 probes. (B) WT and *Clec4d* ^*-/-*^ BMDMs were stimulated with *C*. *tropicalis* (MOI = 1) or LPS (100 ng/ml) for the indicated times. Cell lysates were subjected to immunoblot analysis using the indicated antibodies. (C) BMDMs from WT and *Clec4d* ^*-/-*^ mice were stimulated with *C*. *tropicalis* for the indicated times. Cell lysates were collected and subjected to immunoprecipitation using anti-CARD9 antibodies. The precipitates and lysates were subjected to immunoblot analysis using the indicated antibodies. (D) WT and *Clec4d* ^*-/-*^ BMDMs were stimulated with *C*. *tropicalis* for 12 h. The production levels of IL-6, TNF-α, IL-10, and IL-1β in the supernatants were assayed using ELISA. Data represent one of three independent experiments. Error bars, SD. *P <0.05; **P < 0.01.

Our previous data has proved that trehalose 6,6'-dimycolate (TDM)-induced Mincle expression is dependent on Dectin-3-mediated NF-κB activation via CARD9-BCL10- MALT1 complex [23]. This result prompted us to investigate whether Dectin-3 contributes to the NF-κB activation following challenge with *C*. *tropicalis*. Indeed, both hyphae and yeast form of *C*. *tropicalis* stimulation could effectively induce NF-κB activation in wild-type BMDMs (S5 Fig), and it was significantly defective in *Clec4d* ^*-/-*^ BMDMs (Fig 5A). Consistently, IκBα phosphorylation and degradation were partly defective in *Clec4d* ^*-/-*^ macrophages upon *C*. *tropicalis* stimulation (Fig 5B).

Previous studies have shown that CLRs recognize fungi and induce inflammatory responses through the adaptor protein CARD9 [21,26], and CARD9 forms a complex with Bcl10 following *C*. *albicans* stimulation [27]. We then examined the inducible CARD9/Bcl10 complex formation upon *C*.*tropicalis* stimulation, and found that the formation of this complex was defective in *Clec4d* ^*-/-*^ BMDMs following the stimulation of *C*. *tropicalis* (Fig 5C). The expression levels of some NF-κB regulated genes, including IL-6, TNF-α, and IL-10, were markedly lower in *Clec4d* ^*-/-*^ BMDMs than in wild-type BMDMs (Fig 5D). Together, these data indicated that Dectin-3 plays a crucial role in NF-κB mediated inflammatory response to *C*. *tropicalis* infection.

### Dectin-3 is required for tissue repair during fungal invasion

**Fig 6:**
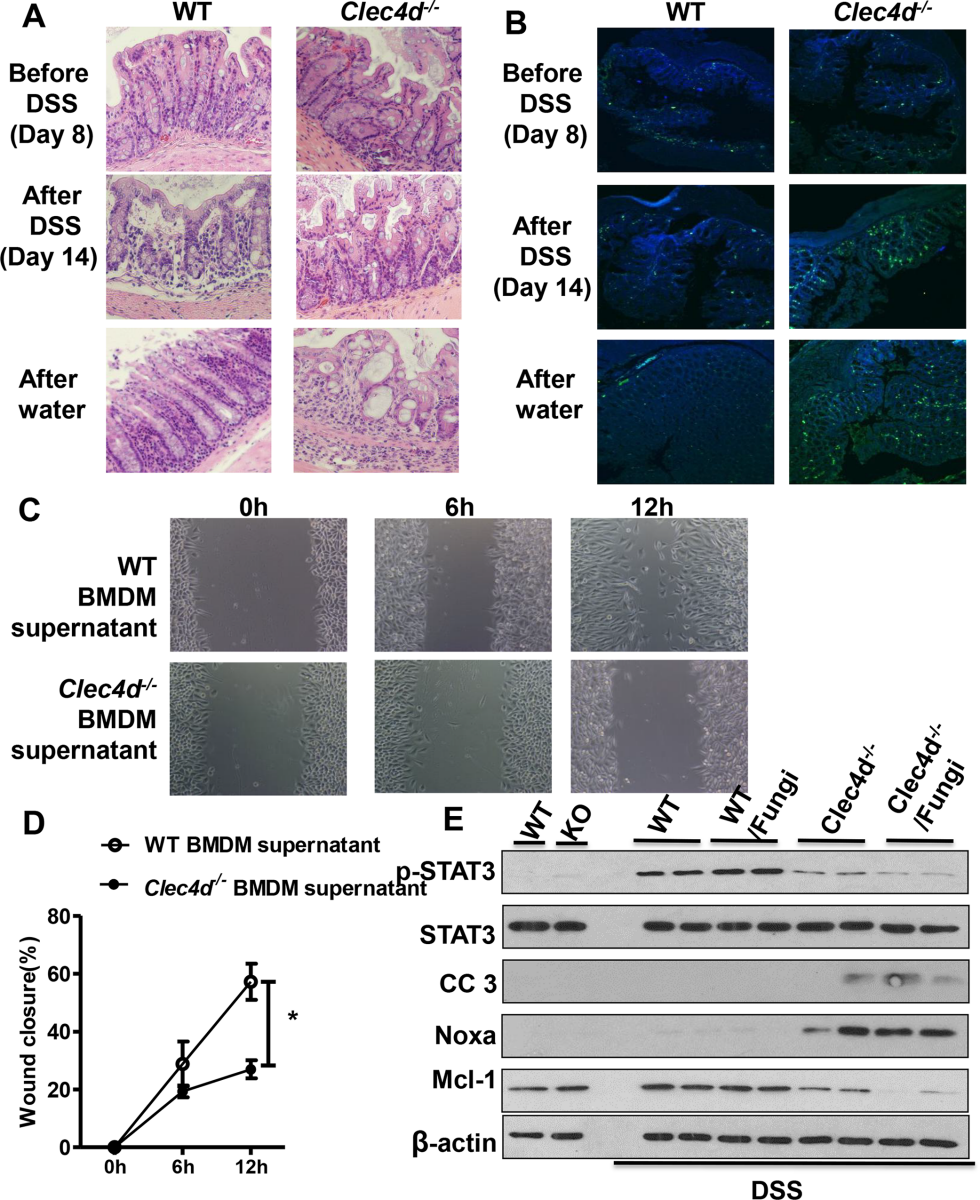
Dectin-3 is required for tissue repair during fungal invasion. (A) WT and *Clec4d* ^*-/-*^ mice (n = 6 per group) underwent supplementation with or without four doses of GFP-labeled *C*. *tropicalis* and then given 2.5% DSS for 7 days and water for an additional 4 days. Mice were killed before treatment with DSS (n = 2, day 8), after the treatment (n = 2, day 14), and after being given water (n = 2, day 18). Colons were examined using H&E staining. (B) Mice were given treatment as described in A. Colon sections were stained with an anti-GFP antibody and counterstained with DAPI. (C-D) BMDMs were acquired from WT and *Clec4d* ^*-/-*^ mice and stimulated with or without *C*. *tropicalis* for 12 h. Supernatant of BMDMs was collected. Migration ability of NCM460 cells were analyzed using a wound healing assay in the presence of 1 ml BMDM supernatant. The wound closure percentage was calculated and analyzed. (E) Mice were treated as described in Fig 3E. Colon cells were separated and lysed, and the lysates were subjected to immunoblot analysis with the indicated antibodies. Data represent one of three independent experiments. Error bars, SD. *P <0.05.

In our DSS-induced colitis model, we found that body weight loss did not differ among wild-type and *Clec4d* ^*-/-*^ mice during DSS treatment stage but did during recovery from treatment (water supplementation stage). Moreover, the defect in IL-6 expression observed in *Clec4d* ^*-/-*^ mice also may play a role in impaired epithelial restitution. These data suggested that Dectin-3 plays a role in intestinal healing. Therefore, we performed histological analysis of colon in the different stages of our fungal supplementation experiments. We found that both wild-type and *Clec4d* ^*-/-*^ mice had colon epithelial cell damage with DSS treatment. After recovery from treatment, most of the epithelial cells in the colons of wild-type mice were repaired while *Clec4d* ^*-/-*^ mice had impaired tissue repair (Fig 6A). Examination of colons of these mice revealed that more fungi invaded damaged tissue in DSS-treated *Clec4d* ^*-/-*^ mice than in wild-type mice (Fig 6B).

Furthermore, we performed a wound-healing assay to examine the tissue repair function in a normal colon epithelial cell NCM460 in vitro. We obtained BMDMs from wild-type and *Clec4d* ^*-/-*^ mice and stimulated the cells with *C*. *tropicalis* for 12 hours. We then collected BMDMs supernatants and added them to NCM460 cells. After 12 h, NCM460 cells cultured with *Clec4d* ^*-/-*^ BMDMs supernatant had decreased cell migration as determined using the wound-healing assay (Fig 6C and 6D) and decreased expression of p-STAT3 (S6A Fig). We also found markedly lower p-STAT3 and Mcl-1 and higher cleaved caspase-3 and Noxa expressions in colon tissues obtained from *Clec4d* ^*-/-*^ mice than in that obtained from wild-type mice (Fig 6E). To determine the nature of the cytokine responsible for tissue repair, IL-6 antibody was added into BMDMs supernatant. As shown in S6A and S6B Fig, IL-6 blocking in WT BMDMs supernatant can inhibit cell migration and down-regulated the expression of p-STAT3 in NCM460 cells. This data suggest that defective activation of NF-κB and IL-6 production lead to an impaired tissue repair of colon tissues and a more severe colitis in *Clec4d* ^*-/-*^ mice.

### Antifungal treatment ameliorates DSS-induced colitis

**Fig 7:**
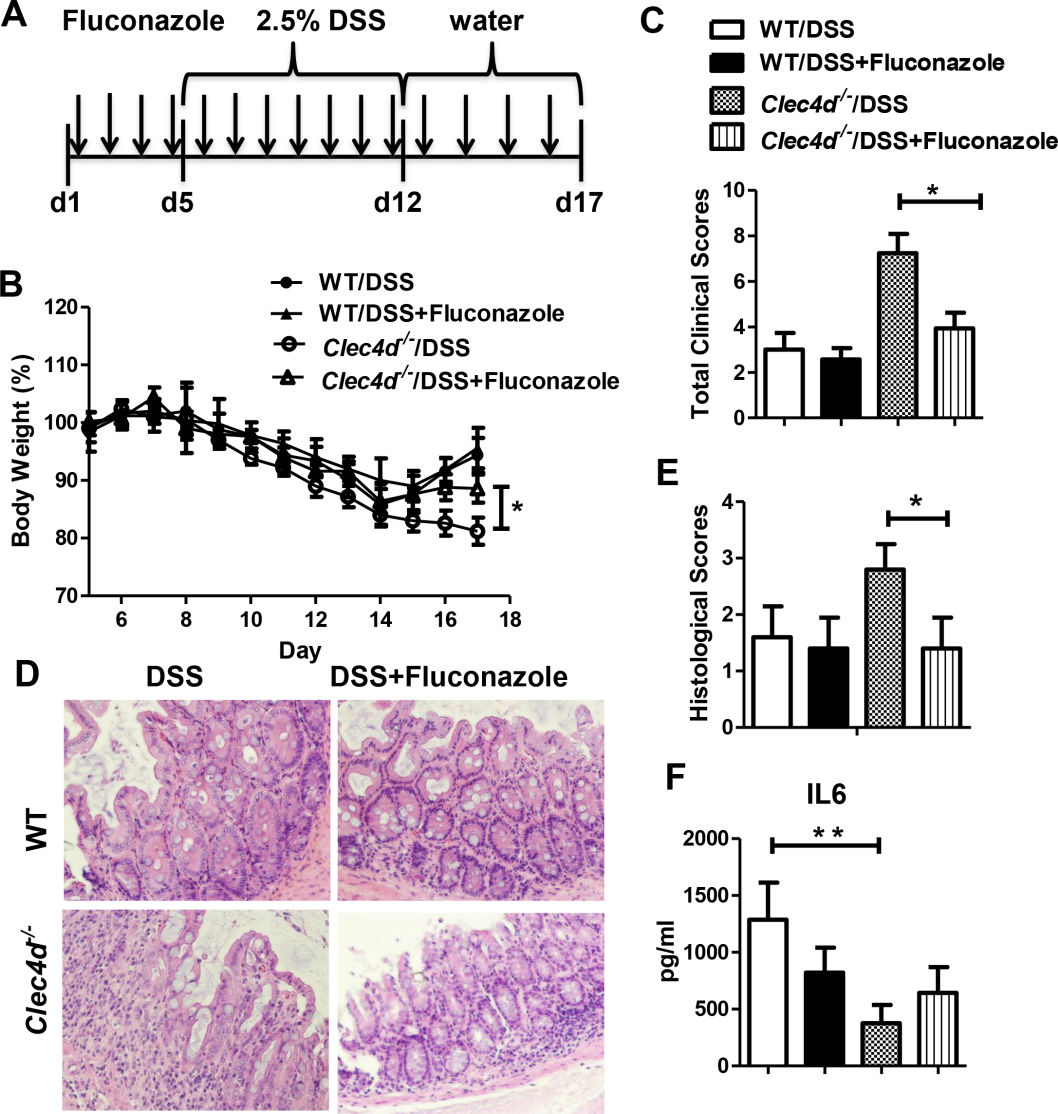
Antifungal therapy ameliorates colitis in Dectin-3-deficient mice. (A) WT and *Clec4d* ^*-/-*^ mice (n = 5 per group) were given with or without fluconazole (0.5mg/ml) during DSS treatment. (B-C) Severity of colitis were assessed by measuring body weight during treatment and examining clinical colitis scores. (D and E) Colons were examined using H&E staining and assigned pathological scores. (F) IL-6 production levels in colonic LP cells were assayed using ELISA. Data represent one of two independent experiments. Error bars, SD. *P <0.05.

To determine whether an altered fungal burden contributes to colitis severity in the absence of Dectin-3 expression, we suppressed fungal growth in mice via treatment with fluconazole as outlined (Fig 7A). Fluconazole treatment could significantly inhibit the proliferation of fungus in *Clec4d* ^*-/-*^ colitis mice (S7A Fig). Although fluconazole treatment could slightly increase bacteria burden in both wild-type and *Clec4d* ^*-/-*^ mice, no significant difference of bacteria burden was found between wild-type and *Clec4d* ^*-/-*^ mice (S7B Fig). The treatment led to reduced weight loss (Fig 7B) and lower clinical and histological scores in *Clec4d* ^*-/-*^ mice (Fig 7C–7E). We also checked the IL-6 expression level and found no difference between wild-type and *Clec4d* ^*-/-*^ mice upon fluconazole treatment (Fig 7F). Taken together, these results further support the conclusion that an inability to control fungi in the gut leads to more severe colitis in Dectin-3-deficient mice.

**Fig 8:**
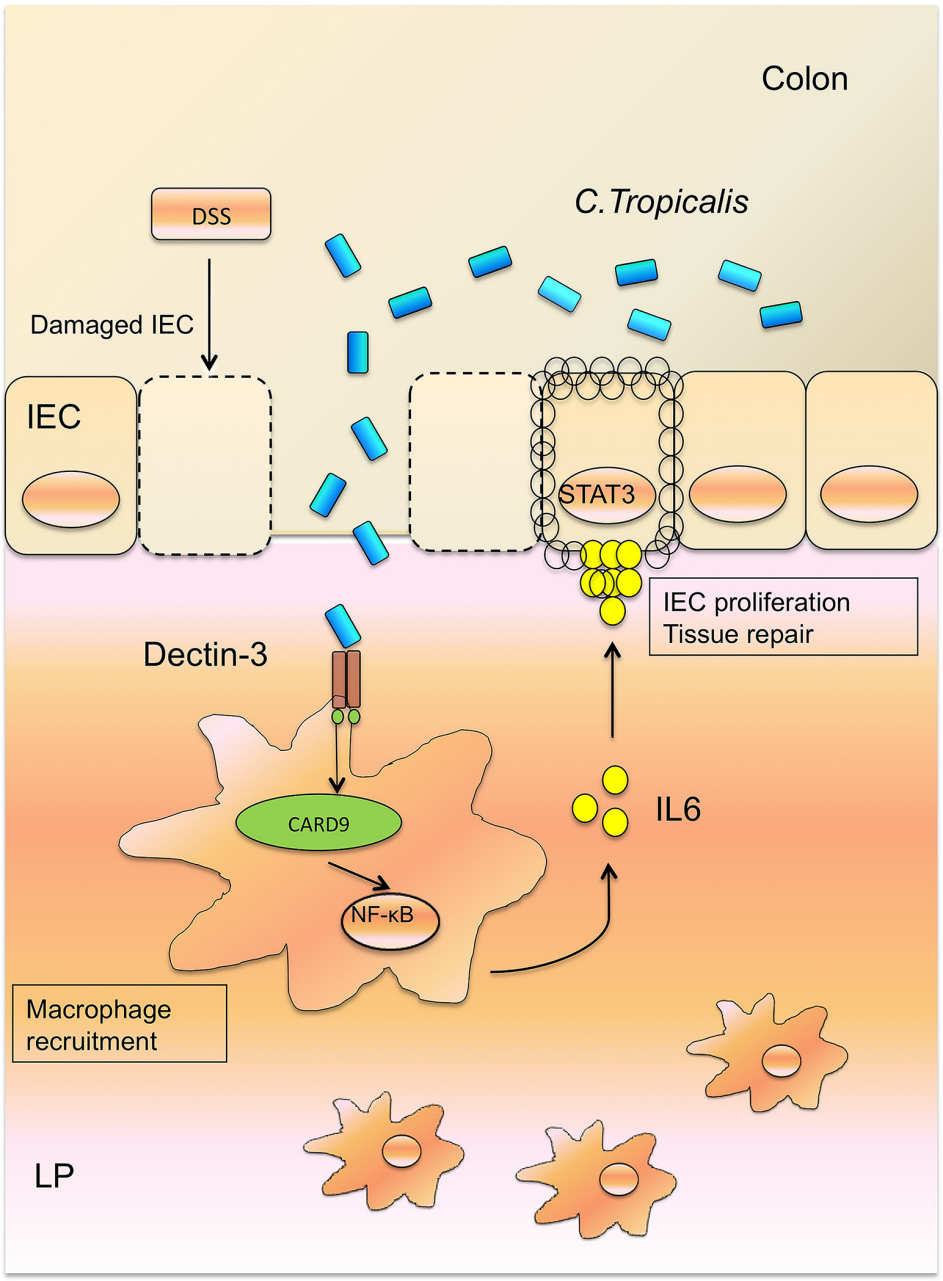
A working model summarizing the findings of this study.

In summary, we proposed our working model as follows (Fig 8): *C*. *tropicalis* is a type of commensal fungus that does not induce colitis under normal situations. After tissue damage induced by DSS, *C*. *tropicalis* translocated to the LP and activated NF-κB signaling via Dectin-3 and CARD9, triggering anti-fungal innate immune responses. In *Clec4d* ^*-/-*^ mice, Dectin-3-dependent NF-κB activation was defective, and IL-6 production was decreased. Loss of these innate immune effector molecules impaired tissue repair, leading to increased microbial translocation and chronic stimulation of mononuclear cells, which exacerbated the vicious cycle of colitis.

## Discussion

Genetic variants that confer susceptibility to IBD in humans highlight the importance of innate immune interactions with intestinal microbiota in both initiating and controlling inflammation. Commensal and pathogenic microorganisms are recognized according to conservation of molecular patterns by pattern-recognition receptors. Herein we describe for the first time that Dectin-3 can recognize *C*. *tropicalis* and is involved in the pathogenesis of colitis. We observed several important findings. First, *C*. *tropicalis* is an opportunistic pathogen, and its burden is specifically increased in *Clec4d* ^*-/-*^ mice during induction of colitis. Second, *C*. *tropicalis* can induce NF-κB activation and cytokine production via Dectin-3 signaling. Third, *Clec4d* ^*-/-*^ mice is more susceptible to DSS-induced colitis than wild-type mice, and *C*. *tropicalis* aggravates the development of colitis.

In mammals, the gastrointestinal tract is colonized by a wide range of microorganisms. Colonization by some commensal or pathogenic microorganisms can be detrimental, leading to infectious diseases. Different commensal microorganisms do not necessarily share the same mechanisms of IBD induction. For example, 129S/SvEv IL10^-/-^ mice associated with either Escherichia coli or Enterococcus faecalis have different clinical signs of IBD [28]. In our Dectin-3 knockout mice, increased burden of *C*. *tropicalis* was the main cause of severe colitis. In the data by Iliev et al., it was also shown that *C*.*tropicalis* was the dominating species in Dectin-1-deficient mice upon DSS treatment [9]. Transplantation of feces from wild-type to Dectin-1 deficient mice did not reduce symptom severity implying that disease severity was host-mediated rather than owing to microbe dysbiosis. In our study, the basal level of total fungal burden in feces was similar between *Clec4d* ^*-/-*^ and wild-type mice. But after DSS-treatment, the total fungal burden in the colon was markedly higher in *Clec4d* ^*-/-*^ mice than that in wild-type mice. These results suggest that the disease phenotype in *Clec4d* ^*-/-*^ mice is affected by the genotype of the mouse, not by initial differences in microbe. However, the role of Dectin-3 in fungal defense is not specific to *C*.*tropicalis*. Our previous study has proved that Dectin-3 can form a heterodimer with Dectin-2 and recognize *C*.*albicans* hyphae. Here we focus on *C*.*tropicalis* due to the dominate role of *C*.*tropicalis* in gu*t*. We did not try gavaging *C*. *albicans* or *C*. *glabrata* in our study. Together, these data suggest that CLR (both Dectin-1 and Dectin-3) deficiency leads to altered immunity to commensal fungi in the gut.

The unique microbial environment of the intestines makes the innate immune system central to intestinal homeostasis. This system is not simply a host-defense mechanism against invading pathogens, as it also modulates microbial killing and affects IEC proliferation, differentiation, and survival. A balance between cell death and survival is important for the maintenance of intestinal homeostasis. NF-κB, a master transcriptional regulator that is activated by various cytokine and pattern-recognition receptors, controls the expression of pro-inflammatory mediators and enhances the survival of cells by inducing the expression of anti-apoptotic genes during colonic inflammation [29]. In mice deficient in the NF-κB p50 subunit, the colonic inflammation becomes persistent [30]. Similarly, mice with IEC-specific deletion of the NF-κB component RELA exhibit increased susceptibility to chemically induced colitis [31]. In the present study, we found that after *C*. *tropicalis* stimulation, induction of the expression of pro-inflammatory cytokines, such as IL-6 and TNF-α, was defective in *Clec4d* ^-/-^ mice but not in WT mice. IL-6 produced by immune cells is a key cytokine in antifungal immunity and tissue repair [25], leading to induction of Th17 cell differentiation. Th17 cells are T helper cells that were characterized relatively recently and play major roles in host defense against fungal infections [32]. In consistent, we also found decreased numbers of Th17 cells in the colons and mesenteric lymph nodes of mice.

After epithelial damage, several pathways function in a coordinated manner to restore homeostasis. Cytokines and chemokines are secreted by epithelial and immune cells, which recruit more immune cells to the site of injury and induce cellular proliferation. Specifically, the inflammasome/caspase 1/IL-18/IL-18 receptor/Myd88 axis mediates tissue repair in the intestines [33]. IL-18 binds to the IL-18 receptor, which is expressed by myeloid cells in the lamina propria, and signals through the adaptor Myd88. If this innate immune signaling pathway is impaired (as observed in mice deficient in caspase 1, NLRP3, IL-18, IL-18 receptor, or Myd88), persistent tissue damage leads to translocation of commensal microorganisms to the sub-mucosa, where they stimulate immune cells. Secretion of cytokines by activated immune cells results in IEC apoptosis and chronic intestinal inflammation. Studies by Grivennikov et al have proved that IL-6 protects the intestinal epithelium from injury by regulating intestinal trefoil factor and/or AMP secretion [25]. Therefore, the defect in IL-6 expression observed in Dectin-3 deficient mice also may play a role in impaired epithelial restitution. In the present study, we found that Dectin-3–deficient mice had impaired healing of epithelial wounds, which is due to the defective activation of NF-κb and less production of IL-6, indicating an inherent defect in restitution of colon epithelial barrier.

Dectin-1 and Dectin-3, both belongs to CLRs group, have different structures and ligand spectrum. Dectin-1 contains immunoreceptor tyrosine-based activation motif (ITAM)-like motif in the cytoplasmic portion. Dectin-1 was proved to be a β-glucan receptor. It can sense *C*. *albicans*, *P*. *carinii*, *Leishmania infantum*, *Coccidioides posadasii*, *Histoplasma capsulatum* and *Mycobacterium spp*. Dectin-3 does not have any signaling motif in their cytoplasmic domains and, instead, recruit the ITAM-containing adaptor molecule FcRg to transduce signals. The CRD domain of Dectin-3 is also atypical, because it lacks conserved triple motif essential for Ca2^+^-dependent carbohydrate recognition. Dectin-3 has a similar ligand spectrum as Mincle in pathogen recognition and recognizes pathogens with high-mannose type and TDM. Dectin-3-deficient mice are more sensitive to systemic *C*. *albicans* infection than wild-type mice and develop milder inflammation upon immunization with TDM. Dectin-3-deficient mice are also highly susceptible to Klebsiella pneumonia infection and die from septic shock. Both Dectin-1 and Dectin-3 mediated carbohydrate recognition induces phagocytosis of pathogens, NF-kB activation, and proinflammatory cytokine production in macrophages.

As to IBD, both Dectin-1 and Dectin-3 plays an important role in the maintenance of the intestinal microbe. A polymorphism in the gene of Dectin-1 was identified to be strongly linked with a severe form of ulcerative colitis in humans. However, there is no association between human Dectin-3 and IBD based on currently available GWAS databases. Therefore, a further analysis and identification of Dectin-3 mutations in IBD patients would provide a molecular basis to apply anti-fungal treatment as a potential new therapy for some IBD patients. Actually, we are collecting human sample from colitis patients, we will confirm whether genetic variation in Dectin-3 influences susceptibility of IBD in our future experiment.

## Materials and Methods

### Ethics statement

Animal care and experimental protocols were in accordance with the NIH “Guide for the Care and Use of the laboratory Animals”. All animal experiments and procedures were conducted under the protocol and were approved by the Institutional Animal Care and Use Committee at The University of Texas MD Anderson Cancer Center (Protocol Number 00000911-RN00).

### Mouse strains

*Clec4d* ^*+/-*^ mice were obtained from the NIH-supported Mutant Mouse Regional Resource Centers (http://www.mmrrc.org). *Clec4d* ^*-/-*^ mice generated as previously described [12] were crossed 5 generations onto C57BL/6J background (96.88%). Progeny homozygous for *Clec4d* ^*-/-*^ and *Clec4d* ^*+/-*^ (wild-type) mice with the same genetic background were bred separately for the experiments, and 8–10 weeks female mice were used. All animals were housed in modified barrier facility at the University of Texas MD Anderson Cancer Center.

### Fungal strains

The *C*. *tropicalis* strain (W4162870) is recovered from a patient with candidemia, and kindly provided by Dr. Sarah L. Gaffen (University of Pittsburgh, PA). A single *C*. *tropicalis* colony was grown overnight at 30°C in yeast peptone dextrose medium. For preparation of the fungal hyphal form, *C*. *tropicalis* was washed, resuspended in complete RPMI 1640 medium, and grown for 3 h. The GFP- *C*. *tropicalis* strain is kindly provided by Dr. Richard Bennett (Brown University, RI).

### DSS-induced colitis and colon histopathology

For DSS-induced colitis model, wild-type and *Clec4d* ^*-/-*^ mice were given drinking water supplemented with 2.5% DSS (MP Biomedicals) for 7 days and then given water for an additional 4 days. For our fungal supplementation experiment, before and upon colitis induction, mice were given four doses of *C*. *tropicalis* (1 × 10^8^ yeast/mouse/dose) every other day. For our fungal ablation experiments, mice were given fluconazole (0.5 mg/ml; Sigma-Aldrich) in drinking water 4 days prior and throughout the DSS and water stages for a total of 17 days. Body weight, stool consistency, and gross blood were checked daily. After mice were sacrificed, their colon lengths were measured. Paraffin-embedded colon tissue samples were sectioned and stained with hematoxylin and eosin (H&E) at the Research Histology Facility at MD Anderson. Colitis severity was assessed by a blinded pathologist using clinical and pathological scores as described previously [34]. Briefly, clinical scores were calculated based on weight loss, stool consistency and occult blood as follows: weight loss: 0 (0–5%), 1 (5–10%), 2 (10–20%), and 3 (>20%); stool consistency: 0 (normal), 1 (soft but still formed), 2 (very soft), and 3 (diarrhea); occult blood: 0 (negative hemoccult), 1 (positive hemoccult), 2 (blood traces in stool visible), and 3 (rectal bleeding). Scoring system for inflammation-associated histological changes in the colon is: 0 (no evidence of inflammation), 1 (low level of inflammation with scattered infiltrating mononuclear cells, 1–2 foci), 2 (moderate inflammation with multiple foci), 3 (high level of inflammation with increased vascular density and marked wall thickening), 4 (maximal severity of inflammation with transmural leukocyte infiltration and loss of goblet cells).

### Isolation of colonic lamina propria (LP) cells

Colonic LP cells were isolated from the study mice as described previously [35] with some modifications. Briefly, colons were isolated, resected, opened longitudinally, washed, and cut into pieces. Intestinal pieces were incubated in a digestion medium consisting of RPMI 1640, 5% FBS, 1.5 mg/ml collagenase type IV (Sigma-Aldrich), 5 U/ml DNase (Roche Diagnostics), and 1% penicillin-streptomycin for 30 min at 37°C with gentle shaking. The cell suspensions were filtered through a mesh and then centrifuged at 1300 rpm. LP cells were used for flow cytometry, western blot, and cytokine analysis.

### Flow cytometry

For surface staining, cells were washed and stained with fluorescent-conjugated antibodies for 20 minutes at 4°C. For intracellular cytokine staining, cells were incubated for 5 hours at 37°C with 50 ng/mL phorbol myristate acetate (Sigma-Aldrich), 1 mmol/L ionomycin (Sigma-Aldrich), and 1 mL/ mL GolgiPlug (BD Biosciences). Surface staining was performed followed by intracellular staining using the BD Cytofix/Cytoperm Kit (BD Biosciences). The following antibodies were used for our analysis (BD Pharmingen): F4/80 (#552958), CD11c (#550261), CD4 (#553729), IL-17A (#560438), IFN-γ (#561040) CD25 (#561038) and FoxP-3 (#560408). Fluorescently labeled cells were acquired on a FACS Calibur flow cytometer (BD Biosciences) and analyzed using FlowJo Analysis Software (Tree Star, Inc, Ashland, OR).

### Fungal burden assay

Feces was collected from study mice and suspended in 50 mM Tris buffer (pH 7.5) containing 1 mM EDTA, 0.2% β-mercaptoethanol (Sigma-Aldrich), and 1000 U/ml lyticase (Sigma-Aldrich). The mixture was incubated at 37°C for 30 min, and fungal genomic DNA was isolated from feces and colons using a QIAamp DNA Stool Mini Kit (QIAGEN) according to the manufacturer's instructions. For evaluation of fungal rDNA in feces, 100 ng of total fecal DNA was used as a template and fungal 18S rDNA was evaluated using quantitative PCR analysis. For detection of specific fungi, qPCR was performed in genomic DNA using fungal-specific primers. Quantitative real-time PCR was performed using SYBR Green with an ABI StepOnePlus system (Life Technologies) with the following primers: 18S rDNA (ATTGGAGGGCAAGTCTGGTG; CCGATCCCTAGTCGGCATAG), *C*. *albicans* (CTGTTTGAGCGTCGTTTC; ATGCTTAAGTTCAGCGGGTAG), *C*. *tropicalis* (TTTGGTGGCGGGAGCAATCCT; CGATGCGAGAACCAAGAGATCCGT), and *C*. *glabrata* (CTGCGCTTAACTGCGCGGTT; TGCGAGAACCAAGAGATCCGTT GC). The total fungal burden was calculated by the ΔCt method and normalized to the weight of the fecal samples and the amount of total DNA used. Relative quantity of the specific fungal burden was also calculated by the ΔCt method and normalized to the weight of the fecal samples. The proportion of specific fungi was the ratio of specific fungal burden to total fungal burden.

### Immunofluorescent staining

For in vivo fungal staining, embedded intestinal specimens were sectioned, mounted on microscope slides, and incubated for 40 min in PBS containing 2% FCS. Intestinal sections were stained with an anti-GFP antibody (ab13970; Abcam). Slides were rinsed with PBS and stained for 5 min with 0.1 μg/ml DAPI (Invitrogen) and overlaid with a mounting medium (VECTASHIELD; Vector Laboratories). Slides were examined using a Zeiss Axio Observer fluorescence microscope. All compared images were collected and processed identically.

### BMDMs preparation

Primary cultures of BMDMs obtained from mice were prepared and purity of macrophages was confirmed using flow cytometry as described previously[27]. Briefly, bone marrow cells were harvested from the femurs and tibias of mice. Erythrocytes were then removed from the cells using a hypotonic solution. Cells were cultured for 7 days in DMEM containing 30% conditioned medium from L929 cells.

### Fungal killing assay and phagocytic assay

GFP-*C*. *tropicalis* (5×10^6^) was reuspended in RPMI 1640 supplemented with 5% fetal bovine serum (FBS) and added onto 1×10^6^ wild-type and *Clec4d* ^*−/−*^ BMDMs, and incubated at 37°C in a 5% CO_2_ incubator for 1 hour. Wells were washed and fresh media containing fluconazole (300 μg/ml) was added. At 6 hours and 18 hours, BMDMs were washed three times with PBS, lysed in water, and *C*. *tropicalis* CFU were photographed and calculated by plating on YPD agar. For phagocytic ability of BMDM experiment, flow cytometry of the GFP fluorescence of wild-type and *Clec4d* ^*−/−*^ BMDMs infected with GFP–C.tropicalis (MOI, 5) for 0, 0.5h, and 1h, respectively, then washed extensively with cold PBS and fixed with 2% paraformaldehyde. GFP^+^ BMDMs were calculated.

### Electrophoretic mobility shift assay

BMDMs were stimulated with *C*. *tropicalis* or LPS, and nuclear extracts of BMDMs were prepared. Five micrograms of the resulting nuclear protein was incubated with a ^32^P-labeled NF-κB (E3291) or Oct-1 (E3241) probe (Promega) for 15 min at room temperature and then subjected to PAGE and exposed to x-ray film.

### Western blotting and immunoprecipitation

Cell lysates from BMDMs were immunoprecipitated with indicated antibody-conjugated agarose. The resulting immunoprecipitates and lysates were subjected to SDS-PAGE and then blotted using indicated antibodies. Phosphorylated IκB kinase α/β (#2697), extracellular signal-regulated kinase 1/2 (#9101), P38 (#4631), IκBα (#9246), and STAT3 (##9145); cleaved caspase 3 (#9664); and P38 (#9212) were purchased from Cell Signaling Technology. Antibodies against IκB kinase α (sc-7218), IκBα (sc-371), Noxa (sc-30209), and actin (sc-8432) were purchased from Santa Cruz Biotechnology. An anti-CARD9 antibody was purchased from Sigma (C7862).

### ELISA

BMDMs were stimulated with *C*. *tropicalis* for 12 h, and BMDM supernatants were collected after stimulation. The ELISA kits for TNF, IL-6, IL-10, MIP-2, IL-1β, IL-17a and CXCL1 were purchased from eBioscience. All the supernatant samples were measured in triplicate according to the ebioscience manufacturer’s protocol.

### Wound healing assay

Wounded-monolayer NCM460 cells were washed two or three times to remove detached cells. The initial size of the wound on the monolayer was determined using inverted microscopy immediately after the cells were washed. After 6 and 12 h of incubation in the BMDMs supernatants stimulated with *C*. *tropicalis*, wound closure was calculated as the percentage of the remaining initial wound area.

### Statistical analysis

An unpaired Student *t*-test was used to evaluate differences between experimental groups. Statistical analysis was performed using the Prism software program (version 5.0; GraphPad Software). P<0.05 was defined as statistical significance.

## Supporting Information

S1 Fig Related to Fig 1, Dectin-3-deficient mice did not develop spontaneous colitis. (A and B) Body weights were check on female and male wild-type (WT) and *Clec4d* ^*−/−*^ mice (n = 5 per group) on indicated weeks. (C) After 20 weeks, colons were got from WT and *Clec4d* ^*−/−*^ mice. Histological analysis of colons were developed using hematoxylin and eosin (H&E) staining. (D) WT and *Clec4d* ^*-/-*^ single housed mice (*n* = 5 per group) were given 2.5% DSS for 7 days and then water for an additional 4 days. The progress and severity of colitis in the mice were assessed by measuring body weight during treatment. (E) Mice were co-housed for 2 weeks before DSS treatment. Body weight were measured during DSS treatment. Error bars, SD. *P < 0.05.
(TIF)

S2 Fig Related to Fig 2, Dectin-3-deficient mice showed impaired immune responses after DSS administration. WT and *Clec4d* ^*−/−*^ mice were treated as described in Fig 1. (A) Mice (n = 5 each group) were sacrificed in colitis induction stage (Day 7). Expression levels of colonic IL-6, TNF-α, IL-17a, and MIP-2 were detected in WT and *Clec4d* ^*−/−*^ mice. Untreated mice (n = 5 each group) and DSS-treated mice (n = 5 each group) were sacrificed after water recovery stage (Day 13). (B) Cytokine production levels of IL-6, TNF-α, IL-17a, and MIP-2 by colonic LP cells were assayed using ELISA. (C) The colonic expression of IL-10, IL-22, IL-1β and IFN-γwere detected using qPCR. (D) Expression levels of IL-6, TNF-α, IL-17a and MIP-2 in serum were detected using qPCR. (E) Expression levels of IL-6, IL-17a and CXCL1 in MLNs were detected using qPCR. Data represent one of two independent experiments. Error bars, SD. * *P*<0.05, ** *P*<0.01.
(TIF)

S3 Fig Related to Fig 3, supplemental of *C*. *tropicalis* aggravate colitis in Dectin-3 deficient mice. WT and *Clec4d* ^*−/−*^ mice (n = 5 each group) were treated as described in Fig 1. (A) Basal fungal burden of feces in WT and *Clec4d* ^*−/−*^ mice before DSS treatment were detected using qPCR. (B) Ratio of *C*.*albican and C*.*glabrata* to total fungal burden were assayed in the feces of WT and *Clec4d* ^*−/−*^ mice after DSS treatment using qPCR. (C) DNA was isolated from feces of WT and *Clec4d* ^*−/−*^. Quantitative analysis of total bacteria burden was detected using qPCR. (D) WT and *Clec4d* ^*−/−*^ mice (n = 5 per group) were treated as described in Fig 3E. Total fungal burden of feces were detected on day 0, day 8, and day 18 using qPCR. (E and F) WT and *Clec4d* ^*−/−*^ mice (n = 5 per group) were treated as described in Fig 3E. Disease severity was accessed by hematoxylin and eosin (H&E) staining and was calculated by histology score. Data represent one of two independent experiments. Error bars, SD. *** *P*<0.01.
(TIF)

S4 Fig Related to Fig 3, fungi supplemental alone did not induce colitis. (A) WT and *Clec4d* ^*-/-*^ mice (*n* = 5 per group) were given four doses of *C*. *tropicalis* and were kept on water for 14 days. Their body weights were measured during treatment. Colon length (B) and spleen weight (C) were also calculated. Data represent one of two independent experiments. Error bars, SD.
(TIF)

S5 Fig Related to Fig 5, both hyphae and yeast form of *C*. *tropicalis* can induce NF-κB activation. (A) The morphological change of both heat-inactivated and live *C*.*tropicalis* with indicated times. (B) BMDMs from wild type mice were stimulated with hyphae and yeast form of *C*.*tropicalis* for the indicated time points. Nuclear extracts were prepared and subjected to EMSA using 32 P-labeled NF-κB and Oct-1 probes.
(TIF)

S6 Fig Related to Fig 6, Dectin-3 is required for tissue repair during fungal invasion. BMDMs from WT and *Clec4d* ^*-/-*^ mice were stimulated with *C*. *tropicalis* in combined with or without anti-IL6 antibody for 12 h. Supernatant of BMDMs was collected and added to NCM460 cells for indicated times. (A) p-STAT3 and STAT3 expression were detected in NCM460 cells using western blot. (B) Migration ability of NCM460 cells were analyzed using a wound healing assay in the presence of 1 ml BMDM supernatant. The wound closure percentage was calculated and analyzed.
(TIF)

S7 Fig Related to Fig 7, fungal and bacteria burden in feces from WT and *Clec4d* ^*-/-*^ mice. WT and *Clec4d* ^*−/−*^ mice (n = 5 each group) were treated as described in Fig 7A. DNA was isolated from feces of WT and *Clec4d* ^*−/−*^mice after DSS treatment. (A) Total fungal burden were assayed in the feces of WT and *Clec4d* ^*−/−*^ mice using qPCR. (B) Total bacterial burden were assayed in the feces of WT and *Clec4d* ^*−/−*^ mice using qPCR.
(TIF)
