# Supplementary figures and images for: Dectin-3 Deficiency Promotes Colitis Development due to Impaired Antifungal Innate Immune Responses in the Gut
Source: PLoS Pathog. 2016 Jun 9;12(6):e1005662. doi: 10.1371/journal.ppat.1005662 (PMC4900642; doi:10.1371/journal.ppat.1005662)

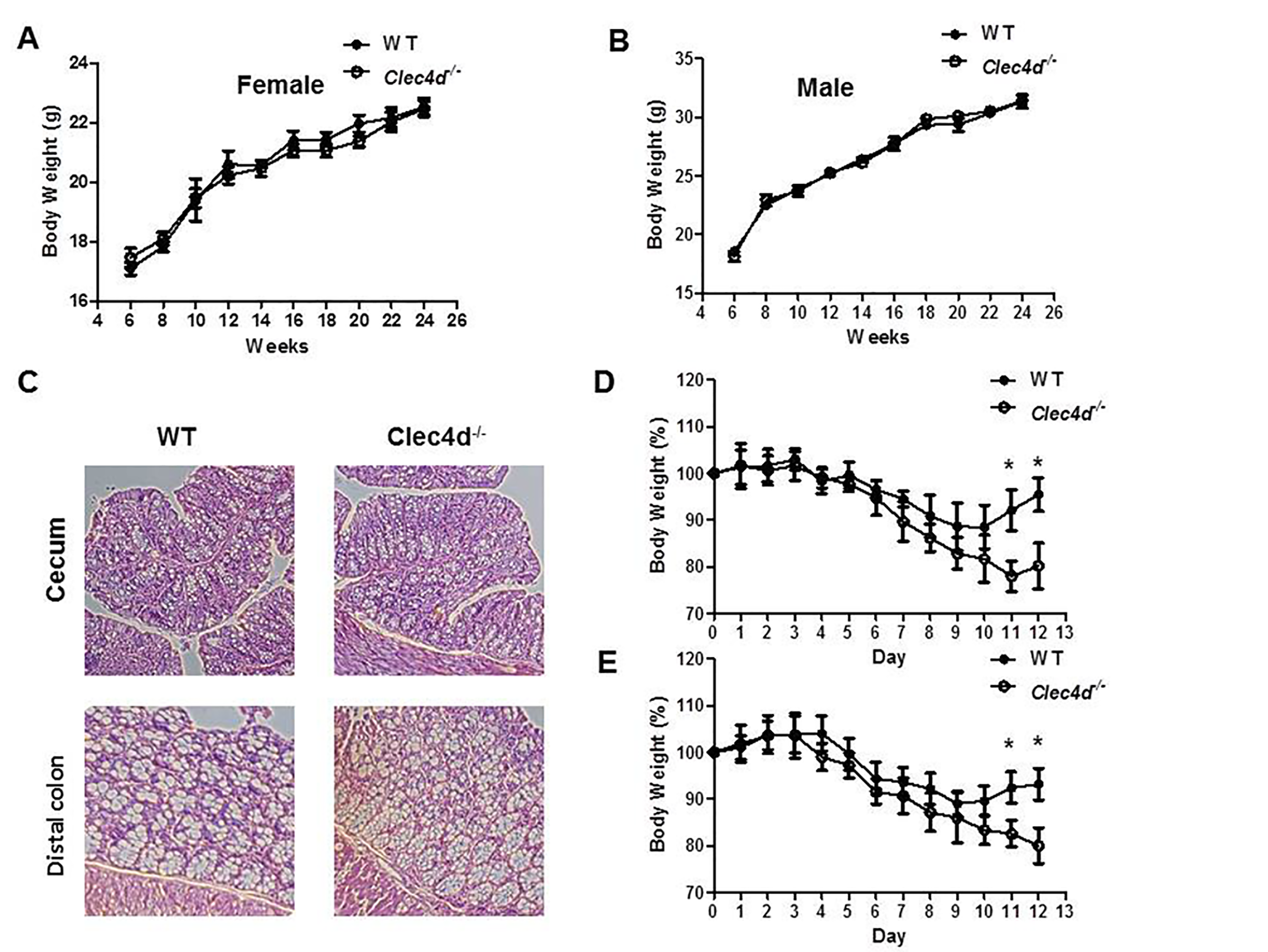

Supplement: S1 Fig — (A and B) Body weights were check on female and male wild-type (WT) and Clec4d −/− mice (n = 5 per group) on indicated weeks. (C) After 20 weeks, colons were got from WT and Clec4d −/− mice. Histological analysis of colons were developed using hematoxylin and eosin (H&E) staining. (D) WT and Clec4d -/- single housed mice (n = 5 per group) were given 2.5% DSS for 7 days and then water for an additional 4 days. The progress and severity of colitis in the mice were assessed by measuring body weight during treatment. (E) Mice were co-housed for 2 weeks before DSS treatment. Body weight were measured during DSS treatment. Error bars, SD. *P < 0.05. (TIF) [file ppat.1005662.s001.tif]

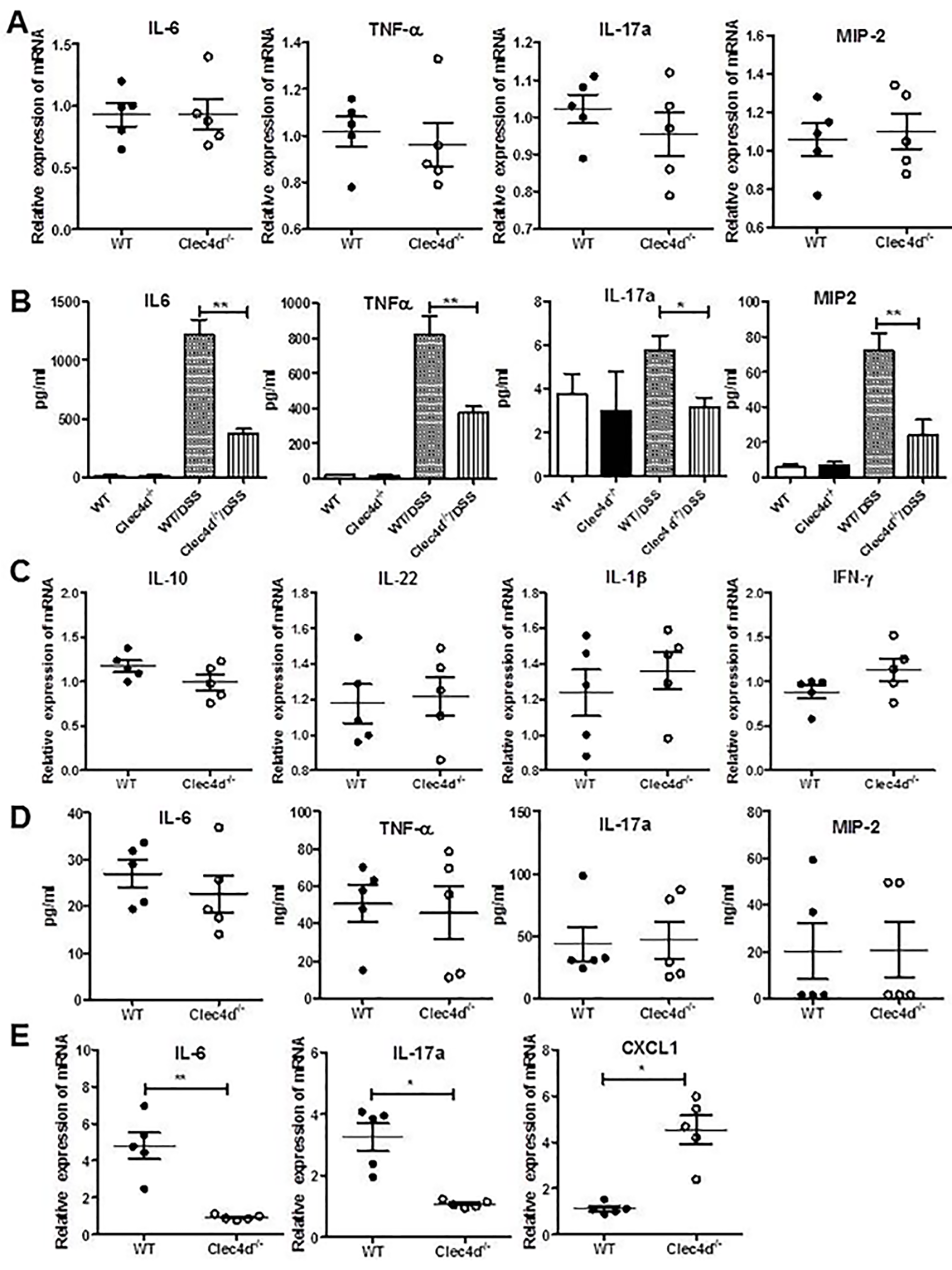

Supplement: S2 Fig — WT and Clec4d −/− mice were treated as described in Fig 1. (A) Mice (n = 5 each group) were sacrificed in colitis induction stage (Day 7). Expression levels of colonic IL-6, TNF-α, IL-17a, and MIP-2 were detected in WT and Clec4d −/− mice. Untreated mice (n = 5 each group) and DSS-treated mice (n = 5 each group) were sacrificed after water recovery stage (Day 13). (B) Cytokine production levels of IL-6, TNF-α, IL-17a, and MIP-2 by colonic LP cells were assayed using ELISA. (C) The colonic expression of IL-10, IL-22, IL-1β and IFN-γwere detected using qPCR. (D) Expression levels of IL-6, TNF-α, IL-17a and MIP-2 in serum were detected using qPCR. (E) Expression levels of IL-6, IL-17a and CXCL1 in MLNs were detected using qPCR. Data represent one of two independent experiments. Error bars, SD. * P<0.05, ** P<0.01. (TIF) [file ppat.1005662.s002.tif]

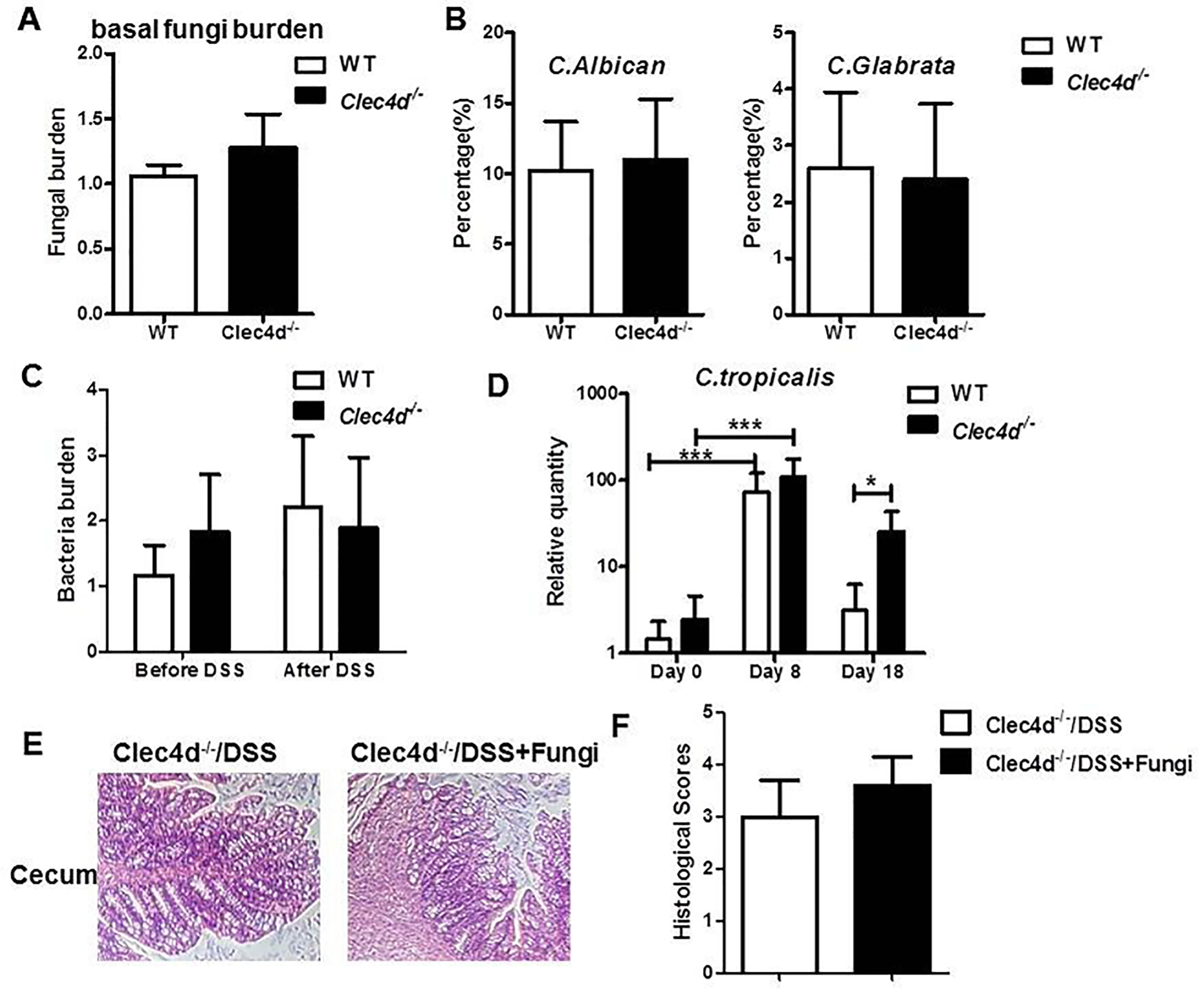

Supplement: S3 Fig — WT and Clec4d −/− mice (n = 5 each group) were treated as described in Fig 1. (A) Basal fungal burden of feces in WT and Clec4d −/− mice before DSS treatment were detected using qPCR. (B) Ratio of C.albican and C.glabrata to total fungal burden were assayed in the feces of WT and Clec4d −/− mice after DSS treatment using qPCR. (C) DNA was isolated from feces of WT and Clec4d −/−. Quantitative analysis of total bacteria burden was detected using qPCR. (D) WT and Clec4d −/− mice (n = 5 per group) were treated as described in Fig 3E. Total fungal burden of feces were detected on day 0, day 8, and day 18 using qPCR. (E and F) WT and Clec4d −/− mice (n = 5 per group) were treated as described in Fig 3E. Disease severity was accessed by hematoxylin and eosin (H&E) staining and was calculated by histology score. Data represent one of two independent experiments. Error bars, SD. *** P<0.01. (TIF) [file ppat.1005662.s003.tif]

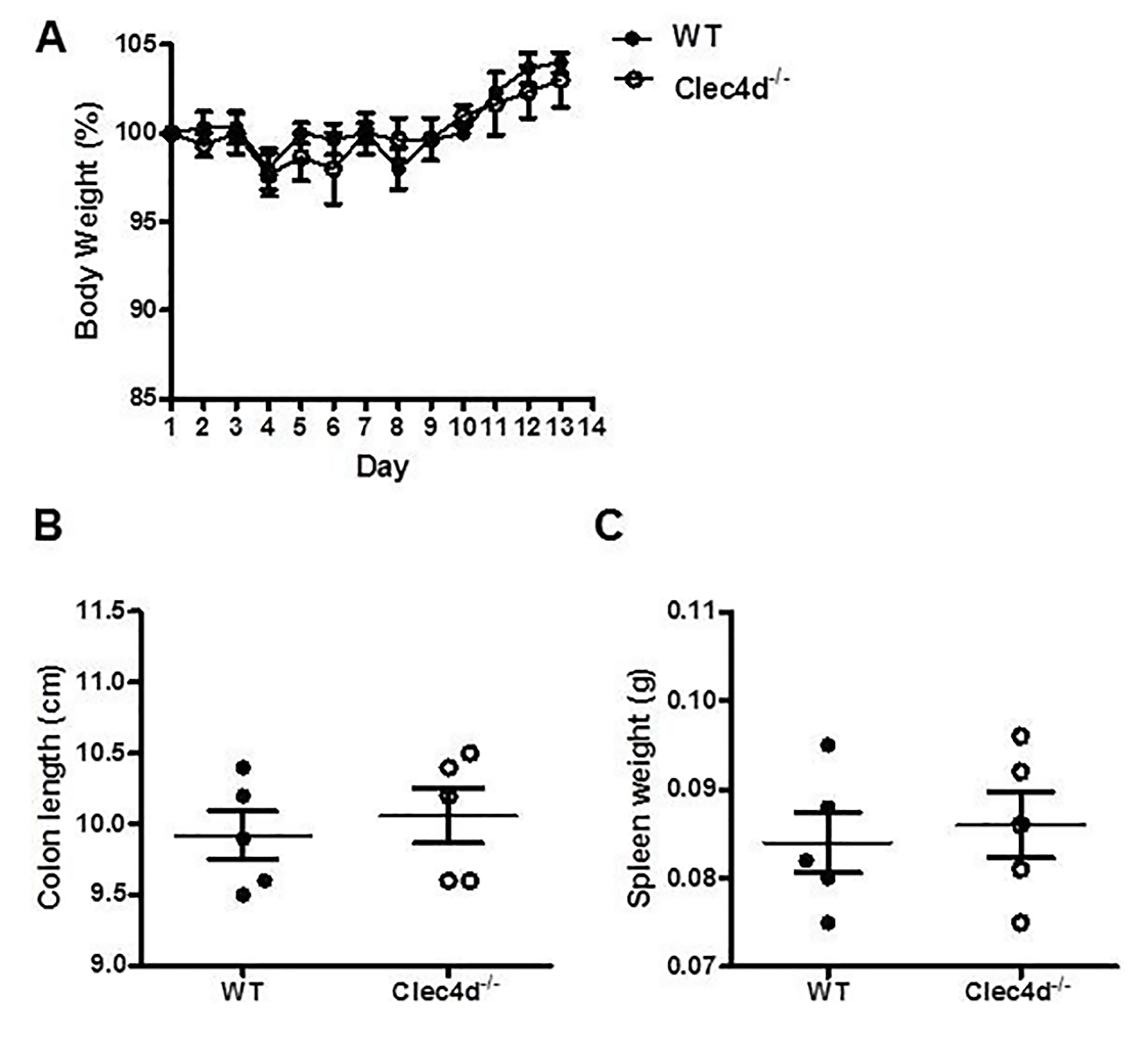

Supplement: S4 Fig — (A) WT and Clec4d -/- mice (n = 5 per group) were given four doses of C. tropicalis and were kept on water for 14 days. Their body weights were measured during treatment. Colon length (B) and spleen weight (C) were also calculated. Data represent one of two independent experiments. Error bars, SD. (TIF) [file ppat.1005662.s004.tif]

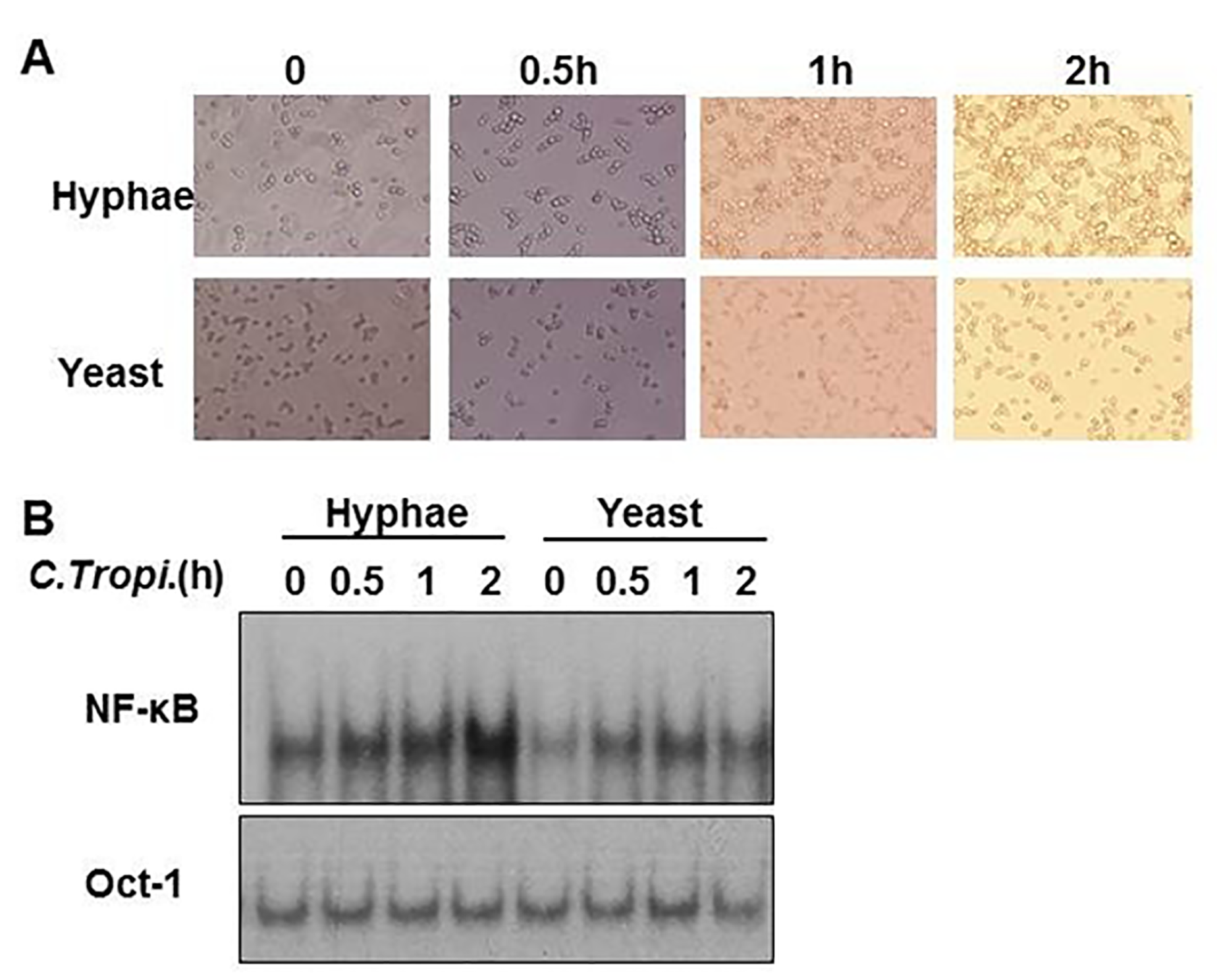

Supplement: S5 Fig — (A) The morphological change of both heat-inactivated and live C.tropicalis with indicated times. (B) BMDMs from wild type mice were stimulated with hyphae and yeast form of C.tropicalis for the indicated time points. Nuclear extracts were prepared and subjected to EMSA using 32 P-labeled NF-κB and Oct-1 probes. (TIF) [file ppat.1005662.s005.tif]

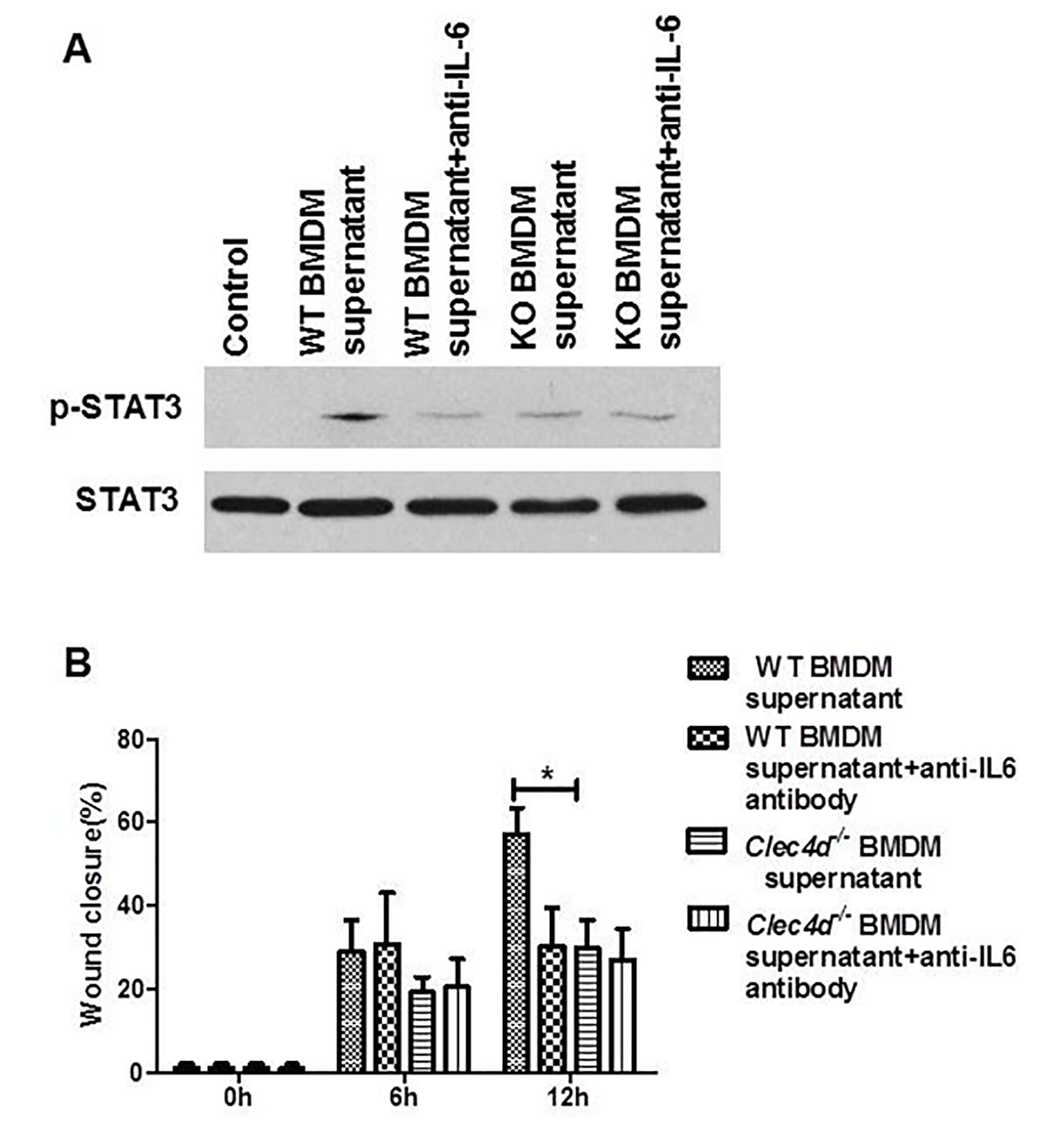

Supplement: S6 Fig — BMDMs from WT and Clec4d -/- mice were stimulated with C. tropicalis in combined with or without anti-IL6 antibody for 12 h. Supernatant of BMDMs was collected and added to NCM460 cells for indicated times. (A) p-STAT3 and STAT3 expression were detected in NCM460 cells using western blot. (B) Migration ability of NCM460 cells were analyzed using a wound healing assay in the presence of 1 ml BMDM supernatant. The wound closure percentage was calculated and analyzed. (TIF) [file ppat.1005662.s006.tif]

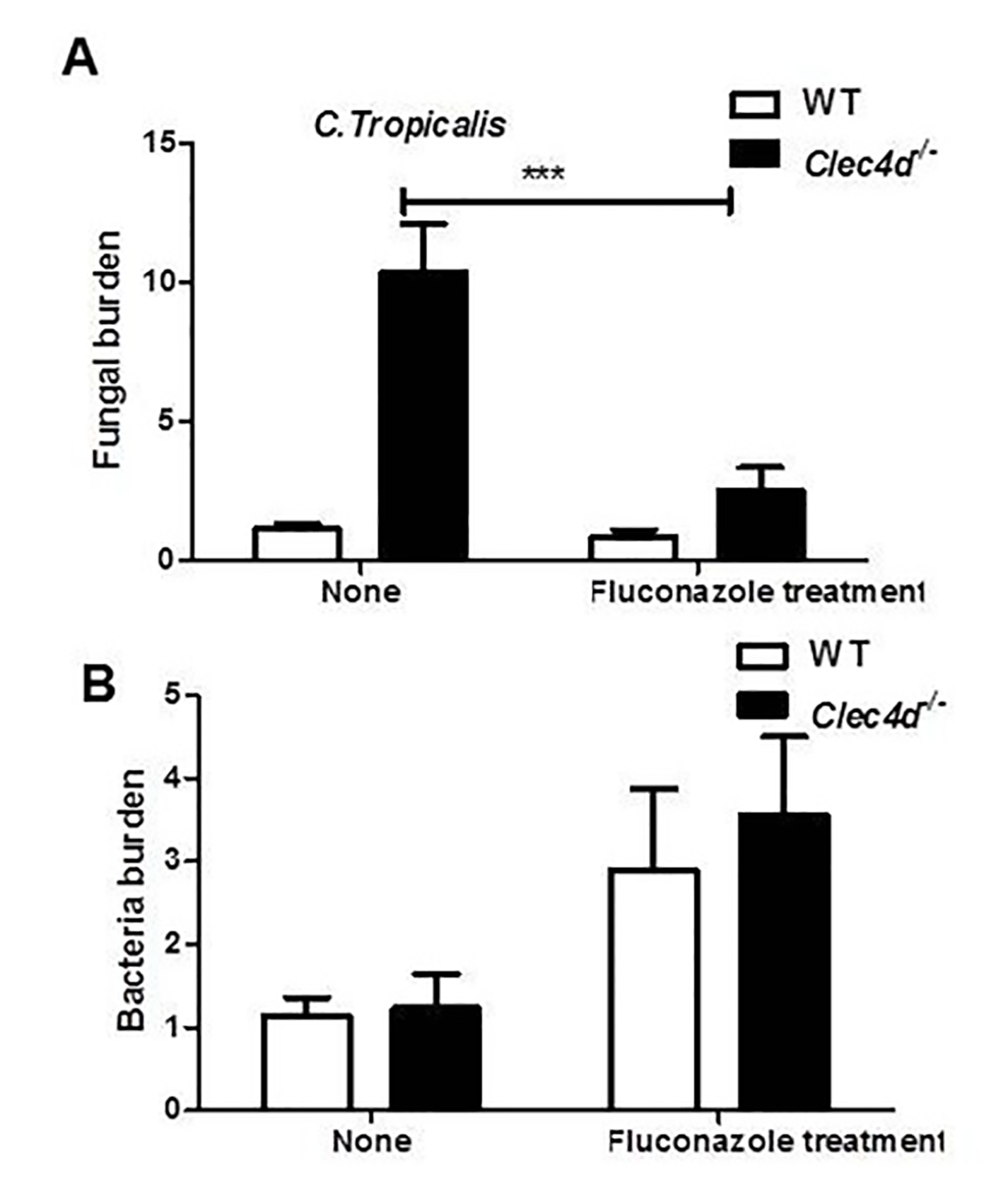

Supplement: S7 Fig — WT and Clec4d −/− mice (n = 5 each group) were treated as described in Fig 7A. DNA was isolated from feces of WT and Clec4d −/−mice after DSS treatment. (A) Total fungal burden were assayed in the feces of WT and Clec4d −/− mice using qPCR. (B) Total bacterial burden were assayed in the feces of WT and Clec4d −/− mice using qPCR. (TIF) [file ppat.1005662.s007.tif]
